# Noninvasive optogenetic induction of cardiac arrhythmias alters systemic hemodynamics in mice

**DOI:** 10.1126/sciadv.aeb1092

**Published:** 2026-06-03

**Authors:** Marcello Magri Amaral, Abigail Matt, Kaelyn H. Schloss, Fei Wang, Elena Gracheva, Yuxuan Wang, Hongwu Liang, Annie Bice, Jimin Ding, Attila Kovacs, Carla Weinheimer, Abhinav Diwan, Jianmin Cui, Stacey L. Rentschler, Jeanne Nerbonne, Christian W. Zemlin, Adam Q. Bauer, Chao Zhou

**Affiliations:** ^1^ Department of Biomedical Engineering, Washington University in St. Louis, St. Louis, MO, USA.; ^2^ Biomedical Engineering, Universidade Brasil, Sao Paulo, Brazil.; ^3^ Department of Radiology, Washington University in St. Louis, St. Louis, MO, USA.; ^4^ Department of Statistics & Data Science, Washington University in St. Louis, St. Louis, MO, USA.; ^5^ Center for Cardiovascular Research, Department of Medicine, Cardiovascular Division, Washington University School of Medicine in St. Louis, St. Louis, MO, USA.; ^6^ Department of Cell Biology and Physiology, Washington University School of Medicine in St. Louis, St. Louis, MO, USA.; ^7^ Division of Cardiology, Medicine Service, John Cochran Veterans Affairs Medical Center, St. Louis, MO, USA.; ^8^ Department of Developmental Biology, Washington University School of Medicine in St. Louis, St. Louis, MO, USA.; ^9^ Division of Cardiothoracic Surgery, Washington University School of Medicine in St. Louis, St. Louis, MO, USA.

## Abstract

Mouse models are valuable for studying the systemic effects of arrhythmia, but traditional methods of heart pacing are invasive and technically complex. Cardiac optogenetics enables precise control of cardiac activity using light-sensitive ion channels and has been used for tachypacing, resynchronization, and defibrillation in animal models. Recent advances in opsins with red-shifted activation spectra and optimized light delivery strategies have enhanced noninvasive pacing approaches, particularly in mammalian models. Here, we use an area illumination approach for light stimulation through the intact chest to perform in vivo, noninvasive optogenetic tachypacing in transgenic mice expressing ReaChR with low irradiance (<1 mW/mm^2^). We assess both cardiac and cortical hemodynamic responses via echocardiography and optical intrinsic signal imaging (OISI). Our findings reveal that whole-heart arrhythmic stimulation alters cortical hemodynamic activity, highlighting the direct impact of arrhythmias on brain perfusion and oxygenation. This work provides insight into the heart-brain connection and the broader systemic consequences of cardiac dysfunction.

## Teaser

Noninvasive cardiac optogenetic pacing was used to alter cardiac and cortical hemodynamics in mice.

## INTRODUCTION

Irregular or abnormal heart rhythms are a leading cause of mortality and morbidity worldwide, affecting ~2.3% of individuals (*1*) and causing 4 to 5 million deaths (*2*) annually. One of the most direct consequences of arrhythmia is disrupted blood flow to vital organs—particularly, the brain—due to irregular beating, altered heart rhythm, chamber desynchrony, and improper chamber filling (*3*). Clinically, patients experiencing atrial fibrillation (AF) report mental fog (*4*) and are at increased risk of stroke (*5*) and cognitive decline (*6*). A study using near-infrared spectroscopy in patients with AF found decreased oxygenation in the frontal and temporal lobes during episodes of AF (*7*). However, cortex-wide oxygenation changes remain unexamined.

Animal models of arrhythmia are powerful tools for studying how irregular heart rhythms alter hemodynamics (*8*). Mice share key cardiac features with humans, including heart structure and development, although they exhibit differences in cardiac action potential morphology and ion channel expression (*8*, *9*). Techniques to assess cerebral hemodynamics in mice, such as optical intrinsic signal imaging (OISI), enable the measurement of oxy- and deoxyhemoglobin changes (*10*). However, sustained arrhythmias and tachypacing using electric pacing methods in mice are technically complex and invasive (*11*, *12*), leaving the effects of arrhythmia on brain hemodynamic changes largely unstudied.

Cardiac optogenetics provides a solution to this limitation by enabling precise, noninvasive control of heart rhythm using light-sensitive ion channels (opsins) (*13*–*20*). First introduced in 2010 (*21*, *22*), cardiac optogenetic techniques have been used to induce tachycardia (*23*, *24*), resynchronization (*24*), and defibrillation (*25*–*27*) in multiple animal models, including *Drosophila melanogaster* (*28*–*30*), zebrafish (*21*), mice (*23*), and rats (*24*, *31*, *32*). Opsins can be delivered to heart tissue by viral vector injection (*23*) or transgenic modification (*22*) and offer advantages such as tissue specificity, high spatiotemporal precision, versatility in stimulation patterns, and the ability to induce arrhythmic activity on demand.

A challenge of mammalian cardiac optogenetics is delivering sufficient light to activate opsins within cardiac tissue. One approach to overcoming this limitation is the use of red-shifted opsins, such as ReaChR (*33*), ChRmine (*34*), Jaws (*35*), and Chrimson (*36*), which respond to light of longer wavelengths that penetrate deeper in tissue. ReaChR, with a peak activation at 590 nm extending up to 630 nm (*33*), has been especially promising for transthoracic cardiac stimulation and has been successfully applied in vivo in *Drosophila* (*29*, *37*) to terminate AF in rats (*38*) and to characterize optogenetic stimulation in Langendorff-perfused mouse hearts (*26*). ChRmine has also been used for transthoracic light stimulation in a study of the connection between the heart and anxiety-like behaviors in mice (*39*).

The optimization of light delivery further enhances optogenetic control. The direct implantation of light-emitting diode (LED) sources onto the heart surface enables precise light targeting but requires invasive surgical placement (*32*, *40*, *41*). Transthoracic illumination using LED point sources on the skin’s surface limits invasiveness but requires high power densities (>100 mW/mm^2^), posing risks of skin burns. An alternative approach involves the use of LED arrays, where each LED point source acts as an individual light source, with diffusive photons from each source cumulatively achieving a greater irradiance. In 2023, Nyns *et al.* (*38*) demonstrated transthoracic pacing and termination of AF in adeno-associated virus (AAV)–injected ReaChR-expressing rats using an LED array with a power density of (~25 mW/mm^2^), well below the American National Standards Institute (ANSI) maximum permissible exposure (MPE) for the skin (~61.9 mW/mm^2^ for a 100-ms pulse of light at 590 nm) (*42*).

Here, we develop and characterize an in vivo model of optogenetically controlled cardiac arrhythmia to study arhythmic hemodynamics. Using a transthoracic light delivery that illuminates a broad skin surface area at low irradiance (<1 mW/mm^2^), we successfully induce tachypacing up to 175% of resting heart rate (RHR) and sustain arrhythmias for up to 10 min in transgenic mice with heart-specific ReaChR expression. Echocardiogram analysis provides a detailed assessment of optogenetic pacing effects on cardiac functions, while OISI enables label-free and minimally invasive cortex-wide evaluation of brain hemodynamic changes before, during, and after arrhythmia. Together, this study demonstrates that noninvasive optogenetic induction of cardiac arrythmias reliably alters systemic hemodynamics in mice, providing a powerful tool to study the cerebrovascular consequences of arrhythmia.

## RESULTS

### A transgenic mouse model with stable, heart-specific, uniformly distributed ReaChR expression

**Fig. 1.**
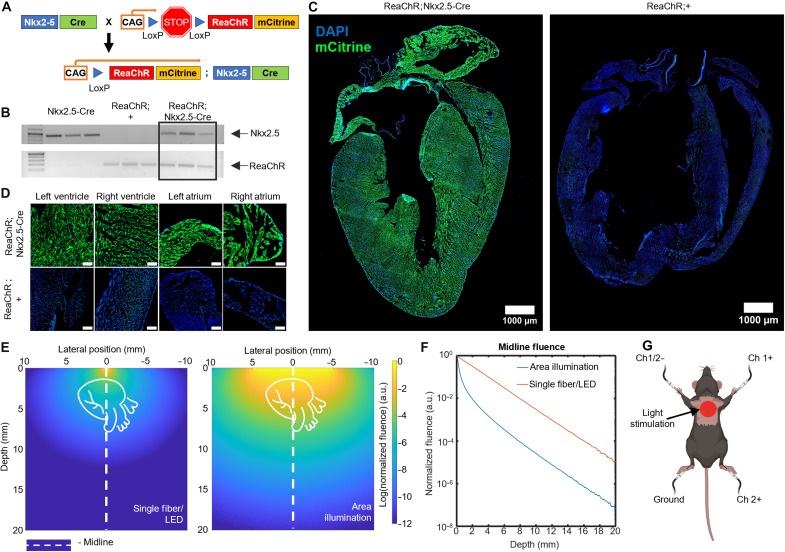
Generation and light stimulation of a ReaChR-expressing mouse strain. (**A**) Schematic of mouse husbandry to generate a mouse with cardiac-specific expression of the ReaChR opsin, where we crossed CAG-LSL-ReaChR-mCitrine (ReaChR;+) mice to transgenic mice with Nkx2.5-regulated Cre (Nkx2.5-Cre). (**B**) PCR results of CAG-LSL-ReaChR-mCitrine/+; Nkx2.5-Cre/+ and CAG-LSL-ReaChR-mCitrine/+; and Nkx2.5-Cre/+ progeny confirmed the presence of both Nkx2.5-Cre and ReaChR genes in the progeny. (**C**) Fluorescence imaging of ReaChR expression in cardiac tissue for a ReaChR;Nkx2.5-Cre and ReaChR;+ mouse. mCitrine fluorescence indicating opsin expression is in green. DAPI-stained nuclei are in blue. Scale bars, 1 mm. (**D**) Zoomed-in fluorescence images of ReaChR-expressing (ReaChR;Nkx2.5-Cre) and ReaChR-mCitrine/+ atrial and ventricular tissue. Scale bars, 1000 μm. (**E**) Results of Monte Carlo (MC) simulations for mouse skin tissue (μ_a_ = 0.9, μ_s_ = 90, *g* = 0.9, *n* = 1.37) scattering for 617 nm were compared for a single fiber/LED illumination and an area illumination (10-mm diameter) light delivery. The heart schematic indicates the representative location of the mouse heart under the skin’s surface. (**F**) Normalized fluence from the midline of the light illumination obtained from the MC simulation in (E). (**G**) Schematic representation of an experimental animal and light positioning and ECG lead positions. Created in BioRender, C. Ren (2026); https://BioRender.com/31stojo.

A mouse line containing the heart-specific Cre recombinase under Nkx2.5 gene control was crossed with a mouse line carrying CAG-LSL-ReaChR-mCitrine transgene in the *Gt(ROSA)26Sor* locus (Fig. 1A). In the obtained progeny, the presence of both CAG-LSL-ReaChR-mCitrine and Nkx2.5 was verified by polymerase chain reaction (PCR) (Fig. 1B). The expression of the ReaChR opsin in the cardiac tissue was confirmed by mCitrine fluorescent marker detection (Fig. 1, C and D). The fluorescent 4′,6-diamidino-2-phenylindole (DAPI) signal (blue) stains the cellular nucleus in both ReaChR;Nkx2.5-Cre and the control group (ReaChR;+). For the ReaChR;Nkx2.5-Cre mice, the mCitrine fluorescence (green) signal shows an even expression of ReaChR opsin throughout the whole cardiac tissue in the four heart chambers (Fig. 1C) and in enlarged tissue images (Fig. 1D). In contrast, no expression (an absence of mCitrine fluorescence signal) is observed in the ReaChR;+ control mice.

The use of optical fibers or a single LED element in direct contact with the skin, a common practice in cardiac optogenetics, produces a small spot size that requires a high irradiance to achieve light pacing (*29*, *39*). Here, we use an area illumination approach to light stimulation, which achieves optogenetic pacing transthoracically with low irradiance. Figure 1E compares the Monte Carlo (MC) simulation results using two light delivery approaches for 617 nm, e.g., a single optical fiber with a 300-μm-diameter versus a 10-mm-diameter area illumination from an LED light source, with the same irradiance level at the surface. The total power reaching 3 mm underneath the surface, e.g., about the depth of the mouse heart from the skin as measured through echocardiogram, is two orders of magnitude greater when the area illumination method is used (Fig. 1F). At the depth of the mouse heart, the spatial distribution of the large illumination area is larger than the spatial distribution of single fiber/LED illumination, and the power distribution is uniform. This makes optogenetic pacing using a large illumination area less sensitive to the illumination position on the skin surface compared to the single fiber/LED illumination method.

The area illumination strategy demonstrated here relies on the superposition of the diffusive photons to achieve higher fluence at the heart surface. A demonstration of this effect can be visualized using a scattering phantom composed of skim milk (fig. S1, A to C), which has a similar scattering coefficient to the biological tissue (*43*, *44*), and the MC simulation for the same scattering phantom (fig. S1, D to F). The wavelength of light also affects its attenuation in tissue. The MC simulation shows that, for the same power density at the skin surface, red light (e.g., 617 nm; Fig. 1, E and F) presents a higher fluence level at the depth of the mouse heart compared to orange light (e.g., 590 nm; fig. S1, G to I). In addition, longer wavelengths penetrate deeper into the biological tissue (fig. S1A) and could represent one order of magnitude higher fluence for larger animals. Our chosen opsin for this study, ReaChR, has peak responsivity at 590 nm, but it also responds to longer wavelengths, such as 617 nm (*33*). A higher irradiance level is obtained with 617-nm light illumination in deep tissue, counterbalancing the reduced responsivity and resulting in both the 590 and 617 nm being suitable for noninvasive transthoracic pacing in ReaChR mice.

In this study, we used ReaChR;Nkx2.5-Cre mice anesthetized with 1.5% isoflurane. We shaved the mouse chest to reduce light attenuation due to the hair. For electrocardiogram (ECG) and echocardiogram experiments, mice were placed in a supine position with a 10-mm light spot size over the chest (Fig. 1G).

### ECG characterization and frequency response during cardiac optogenetic pacing

**Fig. 2.**
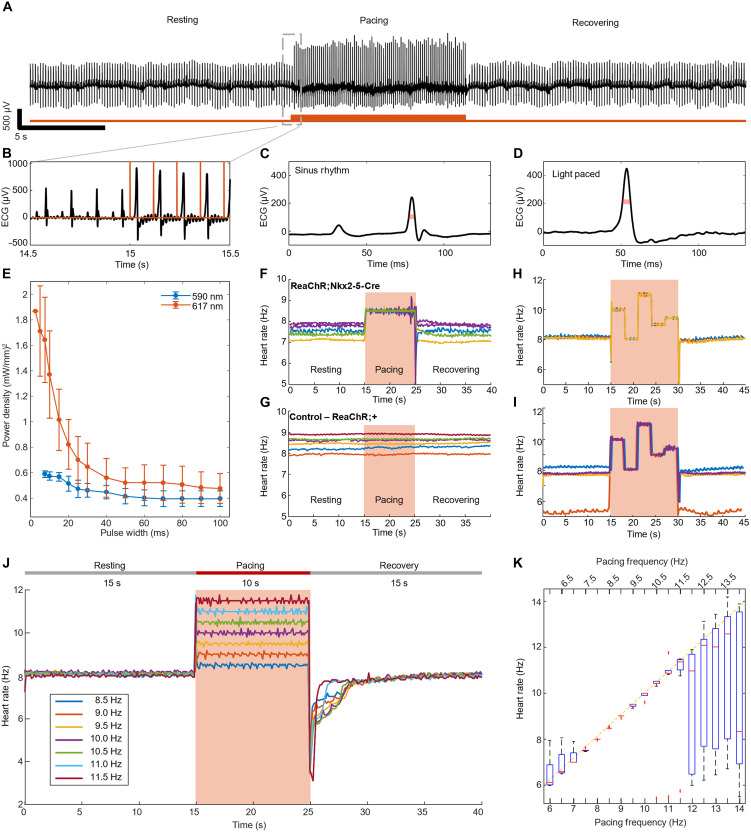
Electrocardiogram validation of optogenetically induced tachycardia. (**A**) ECG recording during an example resting, pacing, and recovering period. Light stimulation was performed with pulses of 20 ms and 1.6 mW/mm^2^ at 8.5-Hz light stimulation (red trace). (**B**) Zoomed-in ECG recording of the transition from resting to light pacing (red trace). (**C** and **D**) Zoomed-in ECG recordings of a single heartbeat due to the sinus rhythm and due to light pacing, respectively. (**E**) Strength duration curves for 590- and 617-nm light (*n* = 11 total, *n* = 3 female, and *n* = 8 male). (**F**) HR during resting, pacing, and recovering periods for multiple different mice in the ReaChR;Nkx2.5-Cre group (*n* = 5 total, *n* = 1 female, and *n* = 4 male), all paced to 8.5 Hz. (**G**) HR during resting, pacing, and recovering periods for multiple different mice the control group (ReaChR;+) (*n* = 6 total, *n* = 4 female, and *n* = 2 male). (**H** and **I**) Variable HR during the pacing interval. The HR during resting, pacing, and recovering periods is shown, with the pacing frequency varied in steps (e.g., 10, 8, 11, 9, and 9.5 Hz). (H) shows different repeats of the same mouse, and (I) shows different mice (*n* = 4 total, *n* = 4 male). (**J**) Representative HR for resting, pacing, and recovering intervals for different pacing frequencies in one mouse (ReaChR;Nkx2.5-Cre). (**K**) HR response for each pacing frequency (*n* = 11 total, *n* = 3 female, and *n* = 8 male). The yellow dashed line plots the linear matching of prescribed pacing frequency to HR response. In the box plot, the box ranges from the 25th to 75th percentile of data. Whiskers extend to the range of data not considered outliers. Outliers are plotted in the red “+” symbols.

To identify changes in cardiac rhythm, lead I and II ECG was recorded with subcutaneous needle electrodes for all ECG characterization experiments, from which the RR intervals were identified and used to measure heart rate (HR) over time. We recorded the ECG signal during resting (15 s), light pacing (10 s), and recovery (15 s). Figure 2A shows the ECG signal during these three periods. A zoomed-in section of the recorded ECG (blue) and light stimulation (red) for 8.5-Hz light pacing is shown in Fig. 2B. This figure reveals that the heart pace changes from a sinus node (SN) rhythm to a light-paced rhythm. The normal QRS complex from the SN rhythm (Fig. 2C) changes to a wider QRS complex initiated after each light pulse (Fig. 2D). The wider QRS complex is consistent with light-induced ventricular pacing, with results achieved by electrical pacing. We used the broadening of the QRS complex (fig. S3) to classify each heartbeat from the ECG recording as captured (initiated by a light pulse) or not captured (initiated by the SN).

The irradiance needed to efficiently pace the heart depends on the opsin sensitivity, wavelength, pulse width, and spot size of the light stimulation. The strength-duration curve shows the minimum optical irradiance at the skin surface needed to efficiently pace the heart at a given pulse width (Fig. 2E). When the mouse’s chest is illuminated with a 10-mm-diameter spot, the strength-duration curves of 590- and 617-nm light show that both wavelengths can efficiently pace the heart with irradiance on the order of a few milliwatts per square millimeter. These irradiance levels are lower than the MPE for a single pulse on the skin (~61.9 mW/mm^2^ for 100-ms pulse duration), and they are more than 100 times lower than the irradiance level used in previous work (~160 to 240 mW/mm^2^) (*29*, *39*). Because of improved light penetration, 617-nm light is comparably effective, which could make it useful for larger animal pacing in the future (fig. S2A). The size of the light spot on the mouse chest can also be adjusted to control the minimum irradiance necessary for safe and effective optogenetic pacing. As shown in fig. S2B, a light stimulation spot size with a 10-mm diameter requires lower irradiance for light capture compared to a 5-mm-diameter spot size. As a result, a 10-mm-diameter spot was used in all the following pacing experiments.

Using this area illumination approach, we demonstrate successful optogenetic pacing in mice in the ReaChR;Nkx2.5-Cre group (*n* = 5; Fig. 2F). All the mouse HRs followed the designed pacing frequency (8.5 Hz), even with different RHRs. For the control group (ReaChR;+, *n* = 5; Fig. 2G), the HR did not change during the pacing period, showing that the presence of the ReaChR opsin is necessary for optogenetic stimulation to occur. In addition, our approach provides full control over the heart rhythm. We demonstrated that the heart rhythm follows the pacing frequency when we change the pacing frequency in steps of 10, 8, 11, 9, and 9.5 Hz for 3 s in each frequency in a 15-s pacing interval (Fig. 2, H and I, movie S1). Figure 2H presents the results of three repeated measurements for the same mouse, where during each trial, the HR will repeatably match the programmed stimulation frequencies. Figure 2I presents measurements for multiple mice (*n* = 4) with different RHRs, where each mouse’s heart can match the programmed light stimulation regardless of their RHR.

Having validated the large area illumination protocol for pacing transgenic ReaChR mice using 617-nm light at low irradiance, we next investigated how the heart responded to different pacing frequencies and how heart functions and hemodynamics changed during pacing. To investigate the response to different pacing frequencies, we paced the heart of ReaChR transgenic mice at frequencies ranging from 6 to 14 Hz while simultaneously recording the ECG signal during resting (15 s), light pacing (10 s), and recovering (15 s) periods.

When paced at frequencies above the RHR (Fig. 2J), the HR follows the light pacing frequency (movies S2 to S4). In all cases, the HR returns to its initial RHR after the pacing period. Figure 2K shows the HR during pacing for different light pacing frequencies (*n* = 11). For frequencies ranging from the RHR (typically between 7 and 8 Hz) to 11 Hz, the measured HR follows the pacing frequency (rho = 0.9958). The use of transgenic animals expressing ReaChR opsin in the heart tissue allows longitudinal studies due to the stability of the opsin expression, and we were able to pace the same animals reliably at different ages, 5 months apart (fig. S4).

### Light tachypacing reduces CO by inducing ventricular tachycardia

**Fig. 3.**
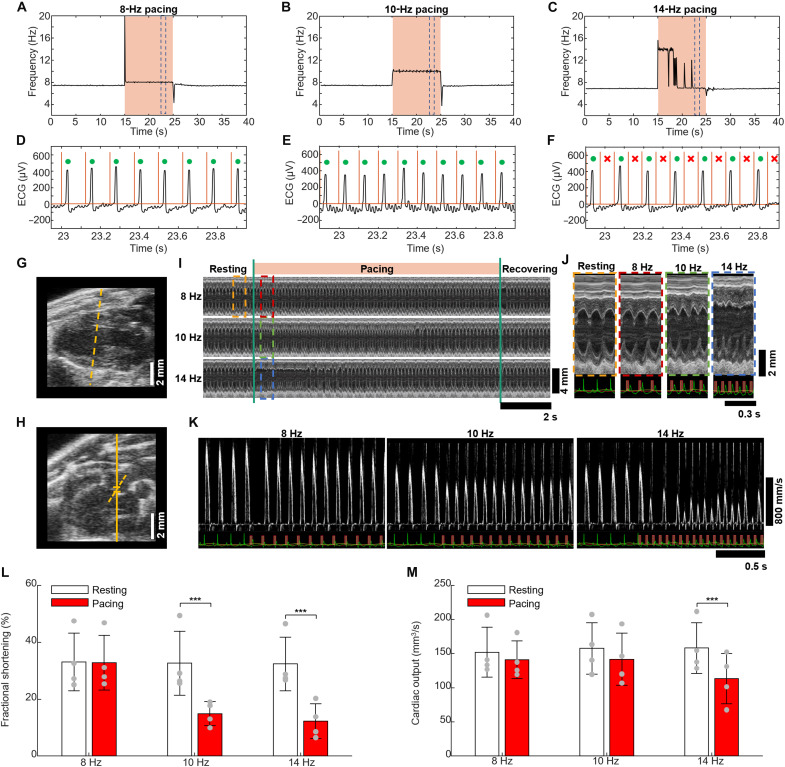
Heart function and hemodynamics characterization for pacing frequencies above RHR. (**A** to **C**) Representative heart responses to light pacing for 8, 10 and 14 Hz pacing. (**D** to **F**) Section of ECG recording and the light stimulation trigger (red trace). The red “X” represents a SN heartbeat, and a green circle reflects a light-captured beat. Orange lines represent the onset of pacing light pulses. (**G**) B-mode echocardiogram image of the long-axis LV. The orange dotted line represents the axis on which the M-Mode images were measured. (**H**) Aortic arch (AA) echocardiogram image. (**I**) M-mode image obtained from the B-mode echocardiogram image presented in (G) for 8-, 10-, and 14-Hz light pacing. (**J**) Zoomed-in detail of M-mode of the LV for resting, 8-, 10-, and 14-Hz pacing, obtained in the dashed rectangle areas in (I). As the pacing frequency increases, an asynchronous contraction between the anterior and posterior sections of the heart wall is observed. (**K**) Blood flow velocity obtained from AA echocardiogram images (H) for 8-, 10-, and 14-Hz light pacing. (**L**) Fractional shortening (FS) for resting and the first second of 8-, 10-, and 14-Hz pacing frequencies, respectively (*n* = 4 total, *n* = 2 female, and *n* = 2 male). (**M**) Cardiac output for resting and the first second of 8-, 10-, and 14-Hz pacing frequencies, respectively (*n* = 4 total, *n* = 2 female and *n* = 2 male). For (L) and (M), a repeated-measures ANOVA followed by a Wald-type *t* test was performed. ***P < 0.001.

Light-induced tachycardia can change cardiac function and hemodynamics. To understand how the heart changes in response to cardiac optogenetic tachypacing, we closely characterized three tachypacing frequencies: 8 Hz (close to the RHR), 10 Hz (close to the threshold for 100% capture), and 14 Hz (above the threshold for 100% capture). At 8 and 10 Hz, the HR can closely match the frequency of light stimulation (Fig. 3, A and B), and every heartbeat is light-captured according to ECG morphology (Fig. 3, D and E). However, at 14 Hz, the rate cannot match the frequency of light stimulation for the 10-s stimulation period (Fig. 3C). Here, the HR initially matches the pacing frequency and jumps to 14 Hz, but, eventually, only every other light pulse induces a heart contraction (Fig. 3F and movie S4). This condition reduces the capture rate to 50% (fig. S5) and the HR to half of the pacing frequency.

To characterize the effect of light pacing on cardiac function and hemodynamics, we obtained echocardiogram images of the long axis of the left ventricle (LV; Fig. 3G and movies S5 to S7) and the aortic arch (AA; Fig. 3H). From the LV time sequence images, we obtained M-mode images (Fig. 3I) and zoomed-in detail (Fig. 3J), which shows the change in heart contraction over time. The anterior part of the heart, which is at the top of the M-mode image, receives most of the light because it is closer to the chest surface. From the M-mode images, we measured the fractional shortening (FS) in the resting and pacing intervals (Fig. 3L and see fig. S6, A to C for time-dependent analysis). The colored boxes in Fig. 3J show the time positions where the FS was measured. A repeated-measures analysis of variance (ANOVA) showed significant differences between resting and pacing phases during the same recording (*P* < 0.0001). Post hoc tests revealed no significant change in FS for 8 Hz (*P* = 0.92). However, a significant reduction in FS was observed for 10- and 14-Hz light pacing (*P* < 0.0001; Fig. 3L). Here, because of the high stimulation frequency, we observed a desynchronization of the heart contraction between the anterior and posterior segments of the heart muscle (Fig. 3J), which can in part explain the reduced FS during the light-induced ventricular tachycardia (Fig. 3L).

Changes in heart function also affect hemodynamics. Blood flow velocity measured from the AA image (Fig. 3H) is reduced during light pacing (Fig. 3K). Cardiac output (CO), the product of stroke volume and HR, is also reduced during light pacing (Fig. 3M and see fig. S6, D to F for time-dependent analysis). A repeated-measures ANOVA revealed significant differences between resting and pacing phases (*P* < 0.0001). At 8 Hz, when the pacing frequency is close to the RHR, post hoc comparisons showed no significant reduction in CO (*P* = 0.18). During 10-Hz pacing, CO was reduced, but not significantly (*P* = 0.06). At the highest tachypacing frequency, 14 Hz, the increase in light stimulation frequency resulted in a significant decrease in CO (*P* < 0.0001). Here, the heart does not have time to properly fill with blood, resulting in a decrease in stroke volume, which is not compensated for by an increase in HR.

### Light bradypacing reduces CO by sinus rhythm competition

**Fig. 4.**
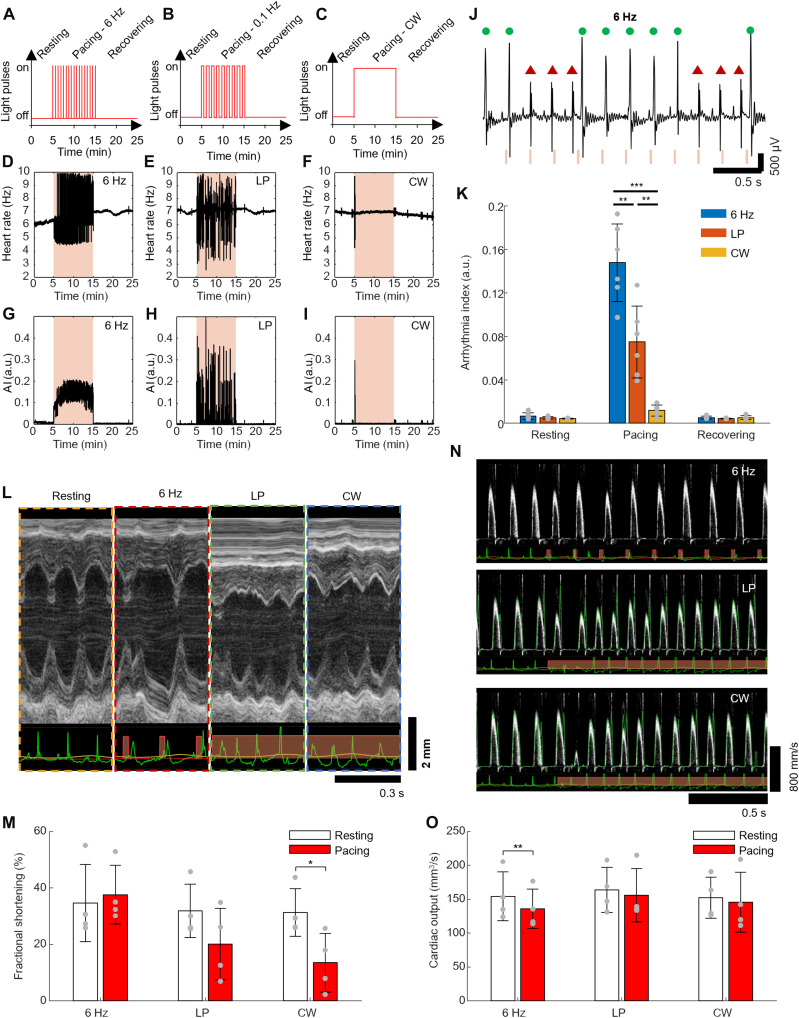
Heart function and hemodynamics for pacing frequencies below RHR. (**A** to **C**) Stimulation protocol for 6 Hz, long-pulse (LP) (5 s pulses - at 0.1 Hz), and continuous illumination (CW). (**D** to **F**) HR frequency over time for the (D) 6 Hz, (E) LP, and (F) CW stimulation protocols. (**G** to **I**) AI over time for the (G) 6 Hz, (H) LP, and (I) CW stimulation protocols. (**J**) ECG recording for the 6-Hz stimulation protocol, showing competition between SN rhythm and light pacing. The red “X” represents a SN heartbeat, and a green circle reflects a light-induced beat. (**K**) Mean arrhythmia index during resting, stimulation, and recovering periods for the 6 Hz, LP, and CW stimulation protocols (*n* = 6 total, *n* = 3 female and *n* = 3 male). (**L**) Representative zoomed-in echocardiography M-mode image during resting, 6 Hz, LP, and CW stimulation. (**M**) FS for resting and the first second of 6 Hz, LP, and CW stimulation (*n* = 4 total, *n* = 2 female and *n* = 2 male). (**N**) Representative zoomed-in blood flow velocity measured over the AA image in the resting to stimulation transition for 6 Hz, LP, and CW stimulation. (**O**) Cardiac output for resting and the first second of 6 Hz, LP, and CW stimulation (*n* = 4 total, *n* = 2 female and *n* = 2 male). For (K), a one-way ANOVA was first performed to confirm differences between groups, and Tukey’s post hoc provided significance values comparing rest and pacing phases. For (M) and (O), a repeated-measures ANOVA followed by a Wald-type *t* test was performed. *P < 0.05, **P < 0.01, and ***P < 0.001.

In the previous sections, we demonstrated that mouse heart function can be noninvasively controlled with a stable frequency during light pacing by choosing the appropriate light-induced tachycardia protocol. We can also obtain highly arrhythmic HR, sustained for several minutes, by pacing the heart with a frequency lower than the RHR. With the goal of creating sustained variability in the RR interval, we stimulated the heart for 10 min with three different protocols: 6 Hz (20-ms light pulse), 0.1 Hz [long pulses (LPs), 5-s light pulse], and continuous illumination [continuous wave (CW)], as shown schematically in Fig. 4 (A to C).

Both the 6 Hz and LP protocols induced and sustained an arrhythmia throughout the 10-min stimulation interval (Fig. 4, D and E). For the 6-Hz stimulation protocol, the longer time between adjacent light pulses created competition between the SN rhythm and light pacing (Fig. 4J and fig. S7A). This competition increased the HR variability (Fig. 4D) and consequently the arrhythmia index (AI) (Fig. 4G). Similarly, the LP protocol also induced a sustained arrhythmia. During LP light stimulation, the heart contraction was dominated by light-induced contraction at the beginning of the 5 s of light illumination, but with a random presence of SN rhythm contraction after a few cardiac cycles (fig. S7B). This behavior increased the HR variability (Fig. 4E) and led to a higher AI during the stimulation interval (Fig. 4H). When the stimulation was constant (CW protocol) throughout the entire 10-min stimulation interval, the HR (Fig. 4F and fig. S7C) initially exhibited more variable HR and an altered AI (Fig. 4I). However, the HR and AI returned to their resting levels after a few cardiac cycles. Although CW illumination does not induce an arrhythmic HR, likely due to light desensitization causing the photocurrent to drop as ReaChR remains open (*33*), it does change the ECG morphology, causing a greater positive deflection after the T wave and negative deflection after the QRS complex (fig. S7, D and E). Comparing the three arrhythmia-inducing protocols (Fig. 4K), the 6 Hz protocol induced the highest mean AI during pacing, followed by LP and CW.

The analysis of the echocardiogram M-mode images from the LV (Fig. 4L) reveals that the FS (Fig. 4M and see fig. S6, G to I for time-dependent analysis) changes during the initial section of the light stimulation for all the protocols. A repeated-measures ANOVA revealed significant differences between resting and pacing phases in both FS and CO (*P* = 0.04 and *P* = 0.007, respectively). During the first second of 6-Hz light stimulation, the FS increases, although not significantly (*P* = 0.67), due to the contributions of longer heart periods where the heart has more time to expand. The blood flow velocity from Fig. 4N shows that the changes in the heart function significantly reduce the CO (*P* = 0.009) due to the reduction in HR (Fig. 4O and fig. S6, J to L for time-dependent analysis).

### Cardiac optogenetic pacing induces cerebral hemodynamic changes

**Fig. 5.**
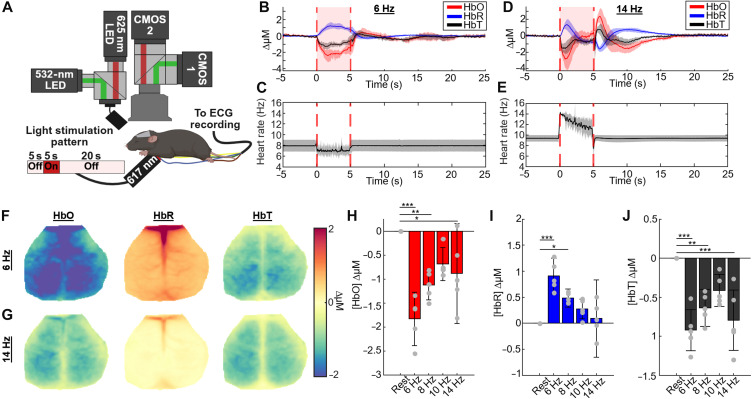
Hemodynamic changes in the brain during optogenetically induced cardiac arrhythmia. (**A**) Experimental setup for concurrent cardiac optogenetic stimulation and OISI recording. Created in BioRender, C. Ren (2026); https://BioRender.com/sundn28. (**B**) Average changes in oxygenated, deoxygenated, and total hemoglobin concentration during 6-Hz light stimulation recordings. Each contrast is plotted as the mean with shaded regions indicating ± SE. (**C**) Average HR during 6 Hz light stimulation recordings plotted as mean HR ± SE. (**D**) Average changes in oxygenated, deoxygenated, and total hemoglobin concentration during 14-Hz light stimulation recordings. Each contrast is plotted as the mean with shaded regions indicating ± SD. (**E**) Average HR during 14-Hz light stimulation recordings plotted as mean HR +/− standard deviation. (**F**) Peak maps showing average changes in oxygenated, deoxygenated, and total hemoglobin concentration across the whole brain during the entire 6-Hz light stimulation period. (**G**) Peak maps showing average changes in concentration of each contrast across the whole brain during the entire14-Hz light stimulation period. (**H** to **J**) Bar plots showing average changes in oxygenated (H), deoxygenated (I), and total (J) hemoglobin concentration during the 10-min rest period before pacing and the entire pacing period in the ReaChR; Nkx-2.5 Cre experimental group. The experimental group reflects *n* = 5 total (*n* = 3 female and *n* = 2 male) mice. Error bars are ±SD. ***P < 0.001, **P < 0.01, and *P < 0.05. A one-way ANOVA was first performed to confirm differences between groups, and Tukey’s post hoc provided significance values comparing rest and pacing phases.

In the above sections, we have shown that light stimulation at a frequency high above or below the heart’s resting rate can result in decreased CO. To determine whether decreased CO affects brain oxygenation, we used OISI to map changes in oxygenated (HbO), deoxygenated (HbR), and total hemoglobin (HbT) concentration over the cortex. Figure 5A shows the experimental setup for cardiac optogenetic experiments with OISI. Mice were fitted with cranial windows and positioned such that most of the dorsal cortex is visible within the OISI field of view. The pacing light source is positioned below the shaved chest of the mouse, and ECG leads were connected to ensure that proper light capture to optogenetic stimulation was observed. Pacing was performed with a 5-s off, 5-s on, and 20-s off cycle for 5 min at a time, resulting in 20 cycles measured for each pacing frequency. In between the 5-min pacing periods, 5 min of rest was observed.

The largest hemodynamic changes in the cortex occurred at the two pacing frequencies farthest away from the RHR: 6 Hz, below the RHR, and 14 Hz, above the RHR. Temporally, during 6-Hz stimulation, the analysis of changes in each contrast over time reveals a sustained effect of cardiac optogenetic pacing, as shown in Fig. 5B (*n* = 5). During the pacing period (0 to 5 s), large and prolonged increases in [HbR] and smaller decreases in [HbO] are observed resulting in a prolonged decrease in [HbT]. Immediately after pacing (5 to 7 s), there is a small overshoot in [HbO] and [HbT] with a recovery to baseline ~10-s post pacing. The prolonged changes in [HbR] during pacing are due to the decreased and more varied HR over this period (Fig. 5C).

Clear differences in dynamics occur over time during 14-Hz pacing (Fig. 5D). Instead of a prolonged decrease in [HbR] as observed in the 6-Hz pacing period (Fig. 5B), there is an initial peak followed by a slow continuous decrease in [HbR], with the converse being true for [HbO]. This peak-decrease [HbR] response can be explained by the initial large increase in HR, followed by a decrease in capture rate over the pacing period (Fig. 5E). Acutely postpacing (5 to 5.1 s), due to conduction blockage, there is a skipped beat resulting in a sharp decrease in [HbO]. Following the skipped beat (5.1 to 7 s), with the return toward normal HR, there is a large overshoot in [HbO] and a smaller undershoot in [HbR], resulting in an overshoot in [HbT]. Following these acute events, the changes in hemoglobin return toward baseline ~15-s postpacing. Temporally, the dynamics of the hemoglobin contrasts are different across pacing frequencies (fig. S8) with the 8- and 10-Hz pacing periods displaying similar peak-plateau dynamics as opposed to the slow and prolonged changes in the 6-Hz pacing group and the faster peak decrease in the 14-Hz pacing group.

Spatially, during the 6-Hz light stimulation period, there is a decrease in [HbO] widely across the cortex, ~1.8 μM (Fig. 5F). Conversely, [HbR] increases in concentration during the light stimulation period, ~0.8 μM over the cortex, with the largest reductions occurring along the sagittal sinus. Because of the larger decrease in [HbO] across the cortex, [HbT] during the pacing period decreases across much of the cortex, except around areas of the sagittal sinus that remain relatively unchanged. Similar spatial trends can be observed during 14-Hz light stimulation, albeit on a smaller scale (Fig. 5G). Videos showing hemodynamic changes over time can be found in movies S8 to S11. Peak maps for recordings with 8- and 10-Hz light stimulation exhibit similar spatial distributions of changes as in recordings with 6- and 14-Hz light stimulation (fig. S9). There are no observed changes in control mice across the entire cortex (*n* = 5; figs. S9 and fig. S10, and movie S12).

In addition, the sagittal sinus and the rest of the cortex exhibit distinct dynamics. Time courses of hemodynamic activity during the pacing period within the sinus (fig. S11A) show that the changes in [HbR] and [HbO] are larger than within the rest of the cortex (fig. S11B). Further, after the pacing period has ended, the overshoot in [HbO] and [HbT] is largest within the sinus and slower to return toward baseline as compared to the rest of the cortex. Similarly to the 6-Hz pacing period, we observe that the sagittal sinus has different spatial and temporal dynamics than the rest of the cortex. The temporal dynamics within the sagittal sinus during the 14-Hz pacing do not show the sharp peak post pacing due to a skipped heartbeat (fig. S11A), athough it is present across the rest of the cortex (fig. S11B). Instead, there is a small dip during the overshoot of [HbO] and undershoot of [HbR]. Further, the post-pacing dynamics are more exaggerated within the sinus; the overshoot then undershoots and returns toward the baseline.

As an additional investigation into temporal dynamics across the cortex, we subdivided the cortex into parcels as per the Allen atlas, where time courses can be found in fig. S10. Each parcel exhibits a decrease in [HbO] in response to pacing, echoing the overall global trend of hypoperfusion, although small differences in the magnitude of these changes exist between parcels.

Figure 5H shows changes in [HbO] at each pacing frequency across the cortex, where 6 Hz shows the greatest decrease, followed by 8, 14, and 10 Hz. [HbR] shows a similar trend (Fig. 5I), although a high SD at 14 Hz drives the average lower than [HbR] changes observed at 10 Hz. Changes in [HbT] across the cortex mimic the trend in the changes in [HbO] across the cortex (Fig. 5J).

## DISCUSSION

We show that noninvasive cardiac optogenetics enables the induction of on-demand, sustained arrhythmias, providing a powerful tool to study the systemic effects of altered hemodynamics on the brain. By using a large illumination area for light stimulation, we achieved transthoracic pacing in mice with low power density, reducing invasiveness and minimizing off-target effects. In addition, OISI allowed for a minimally invasive, label-free assessment of cortex-wide hemodynamic changes. Our results reveal decreases in [HbO] and increases in [HbR] due to optogenetic-induced arrhythmia, particularly along the superior sagittal sinus, indicating widespread cerebral hypoperfusion and disrupted venous flow. These findings show the direct impact of cardiac arrhythmias on brain hemodynamics and highlight the potential of optogenetic approaches to investigate heart-brain connection under both physiological and pathological states.

The reliable induction of the optogenetic arrhythmias by our model allows us to overcome limitations of traditional models of ventricular pacing in mice, which require invasive and technically complex methods to induce. In our study, arrhythmic conditions could be induced on-demand reliably and sustained for up to 10 min (Fig. 4G) through ectopic firing induced by light stimulation, making optogenetics a more promising technique for studies of systemic effects of pacing or long-term pacing in mice. Optogenetics is a targeted approach, meaning that the excitatory effect of light stimulation is confined to the opsin-expressing heart. The use of transgenic animals allows for robust, lifetime expression of the opsin, enabling future longitudinal studies.

We achieved full control of the heart rhythm, tachypacing the heart precisely at designated frequencies during the stimulation interval. During light pacing, the ECG signal shows a broader QRS complex interval and the absence of a P wave, which is typical of light pacing and indicates ventricular pacing (*12*, *39*). However, it is difficult to determine if the P wave is being masked by the QRS complex or if they are combined in the complex. Because of our area light illumination strategy and the fact that ReaChR is expressed evenly throughout heart tissue, it is likely that we are creating a large, light-induced ectopic beat across the ventricle. With this, transthoracic tachycardia could be induced at up to 14 Hz (840 bpm), which is about 75% above the RHR for anesthetized mice. The HR follows the pacing frequency precisely for frequencies ranging from 7.5 to 11.5 Hz (450 to 690 bpm), with a high and sustainable capture rate. Only at the beginning of the stimulation period does a phase mismatch between the SN rhythm and light pacing decrease the capture rate. After this, every one light pulse results in one heartbeat. In addition, arrhythmic conditions and ectopic beats can be created by light pacing above the capture threshold and below the RHR.

At pacing frequencies above the 11.5-Hz capture threshold, the capture rate degrades over time, the onset of which occurs earlier for higher frequencies (fig. S5). The measured off time for ReaChR in human embryonic kidney–293 cells was above 100 ms, and stimulating light pulses in our experiments have a duration of 20 to 30 ms (*33*). Thus, frequencies higher than 10 Hz will cause light pulses to overlap with this off time, potentially causing channels still to be in an open or “closing” state while the next light pulse is shone. Despite the decline in capture rate, we can still create ectopic beats that result in reduced FS and CO. When light is pulsed at 14 Hz, for example, the resultant ECG signal shows a mix of sinus rhythm beats and light induced beats. The analysis of FS and CO over time (fig. S6, C and F) shows an initial sharp decline in the first 5 s of pacing, which is mirrored in the brain by an initial sharp decrease in [HbO] and [HbT] (Fig. 5D). Then, as capture rate declines, FS and CO increase to close to resting state levels. However, in the brain, [HbO] and [HbT] still are decreased below resting state, and after light stimulation is removed, a large overshoot in both contrasts occurs as the brain is reperfused with a proper blood supply.

Pacing the heart at frequencies lower than the RHR creates competition between sinus rhythm and the light pacing rhythm to create a sustained episode of arrhythmic beating. As a result, the HR does not follow the stimulation frequency, but rather adopts a complex rhythm composed of both sinus and light stimulated ectopic beats. Echocardiogram analysis over time showed a sustained decrease in CO during 6-Hz pacing, which in the brain translates to a sustained decrease in [HbO] and [HbT] during the entire pacing period. The 8-Hz pacing, which was below the RHR during OISI studies, also showed a more sustained decrease in [HbO] and [HbT] during the pacing period, although not as strongly as when pacing with 6 Hz (fig. S8B).

OISI provided minimally invasive, label-free measurements of hemodynamic changes on a whole brain scale, revealing cortex-wide decreases in [HbO] and increases in [HbR]. Across the different pacing frequencies, changes in hemoglobin concentration were proportional to the disparity between the pacing frequency and the RHR and the induction of sinus competition or override pacing. Because mice were only lightly anesthetized in this experiment, the RHR is higher than experiments in the above figures (Supplementary Data). An 8 Hz would be a lower pacing frequency than the typical RHR, resulting in the creation of ectopic beats to compete with sinus rhythm. A 10 Hz is above the RHR, albeit not as high above the RHR as in previous experiments.

Spatial and temporal analyses of cerebral hemodynamic changes revealed pacing frequency-dependent changes in [HbO], [HbR], and [HbT] across the cortex. Spatially, light stimulation at all tested frequencies (6, 8, 10, and 14 Hz) led to widespread hypoperfusion across the cortex. This was characterized by a large decrease in [HbO] and a lesser increase in [HbR], resulting in a net decrease in [HbT] across much of the cortex (Fig. 5 and figs. S10 and S11B). However, regions along the superior sagittal sinus showed different spatial patterns. Unlike the broader cortex, [HbT] within the sinus remained relatively stable during the pacing period (fig. S11A). This was due to more balanced changes in decreased [HbO] and increased [HbR], suggesting distinct local hemodynamic regulation or buffering within the venous drainage system.

Temporally, hemodynamic responses varied with pacing frequency. During 6-Hz pacing, below the RHR, there was a sustained decrease in [HbO] and increase in [HbR], resulting in prolonged reduction in [HbT]. Notably, immediately after the pacing period ended, an overshoot in [HbO] and [HbT] was observed, returning to baseline ~10 s postpacing (Fig. 5B). When broken down spatially, this overshoot was more pronounced within the superior sagittal sinus than in the rest of the cortex, and the return to baseline was also slower (fig. S11). Given the sinus’ role as a major venous outflow tract, this delayed recovery suggests that light stimulation at a rate lower than the RHR disrupts venous outflow, leading to impaired clearance and transient accumulation of deoxygenated blood post-stimulation.

These sinus-specific dynamics were consistent but more nuanced at other frequencies. At 14 Hz, above the RHR, there was an initial spike in [HbR] followed by a slow decline, alongside a mirrored decrease in [HbO], reflecting initial override pacing followed by gradual loss of capture (Fig. 5, D and E). A skipped beat immediately following pacing resulted in a sharp dip in [HbO], followed by a large overshoot during recovery. These temporal dynamics were again more exaggerated and prolonged in the sinus compared to the cortex (fig. S11), indicating pacing frequency-dependent differences in venous clearance mechanisms. The sharp dip in [HbO] due to the skipped beat after pacing was not as obvious within the sinus, suggesting local insensitivity to acute cardiac conduction failures. At 8 and 10 Hz, hemodynamic changes were more limited, where temporal profiles exhibited a peak-plateau response during pacing. After 10-Hz pacing, the dip and overshoot dynamics occurred, though on a smaller scale than after 14-Hz pacing.

Overall, the spatial and temporal hemodynamic patterns observed here suggest that the effects of cardiac pacing on cerebral perfusion are multifaceted and depend on both pacing frequency and the brain region. The superior sagittal sinus exhibits distinct dynamics that may reflect its critical role in venous drainage and its unique response to altered cardiac rhythm. These sinus changes demonstrate that optogenetically induced arrhythmia alters global venous hemodynamics. However, as OISI cannot directly quantify blood flow or oxygen extraction, future studies using laser speckle imaging (*45*) could further validate these findings by directly assessing blood flow dynamics.

Our optogenetic arrhythmia model has several advantages over a genetic or acquired disorder arrhythmia mouse model: It is highly configurable in the rate and rhythmicity programmed by light stimulation, noninvasive, and temporally specific, allowing for on-demand activation of arrhythmic heart functioning. The change in hemodynamics can be controlled by adjusting the pacing protocol. Further, our area illumination technique is less sensitive to the positioning of the light spot over the chest than point illumination and could be extended to other optogenetic fields, such as neuroscience, to enable deep tissue stimulation at a lower surface irradiance. In cardiac optogenetics, our illumination strategy, especially when paired with red-shifted excitatory opsins, holds the potential to facilitate pacing in larger animals. This opens previously unexplored avenues for exploration of cardiac physiology, disease mechanisms, and therapeutic interventions using optogenetic tools. The use of noninvasive low irradiance is promising for future clinical uses for transthoracic, noninvasive, and painless optical pacing or cardioversion.

In our current protocol for cardiac optogenetic stimulation, we do not model a clinically significant arrhythmia in our mice. Light-induced heart beats are the result of a more synchronized contraction of the ventricles and reductions in [HbO] likely result from a reduction of blood flow from a heart that was not allowed to fully expand before a light-induced contraction. In humans, ventricular tachycardia can occur from the propagation of excitatory signals through pathways outside of the normal cardiac conduction system. Even hemodynamically stables instances of VT have been shown to decrease cerebral blood flow, leading to hypoperfusion in the brain (*46*, *47*). In addition, it has been shown that cerebral oxygenation decreases during VT in localized regions of the brain (*48*, *49*). In our model, the trend of decreased cerebral oxygenation is conserved on a cortex-wide level, although the conduction of the excitation that drives the tachycardia is not. However, our method of light stimulation and OISI monitoring of cortex-wide hemodynamics could be extended to other cardiac optogenetic models with greater potential for clinical significance. For example, AAVs with atrial specific promoters [ANF (*50*)] or gene painting (*32*, *40*) could be used to further localize cardiac excitation.

In addition, changes in [HbO] and [HbR] alone do not capture the full hemodynamic effects of our optogenetically induced arrhythmia. While OISI reliably measures changes in cortical reflectance, resulting from absorption and scattering changes linked to hemoglobin concentration and oxygenation and tissue scattering, it cannot directly quantify actual blood flow or flow velocity. Reflectance changes are composed of blood volume, blood oxygenation, and light scattering, and separating these contributions precisely is challenging (*51*). Future studies combining OISI with flow-sensitive modalities such as laser speckle imaging or photoacoustic microscopy could provide a more complete picture of how arrhythmic blood flow alterations affect brain perfusion, oxygen delivery, and consumption.

In conclusion, we created a cardiac optogenetic model with robust induced arrhythmia exhibiting physiologically relevant decreases in [HbO] across the cortex. We used ECG, echocardiogram, and OISI to understand electrical and hemodynamic changes due to optogenetically induced arrhythmia. Our use of large area illumination for light stimulation and a transgenic model for whole heart expression of ReaChR allows for low power density control over heart function. This, combined with label-free imaging of whole brain hemodynamics, is minimally invasive to the mouse and could easily be applied to longitudinal studies to further understand the effects of chronic arrhythmia. Furthermore, incorporating genetically encoded calcium indicators in the mouse brain would enable wide-field calcium imaging, providing deeper insights into the neuronal effects of arrhythmia. In addition, our platform could be adapted to study other red light sensitive opsins, such as ChRmine and NpHR, to develop more sophisticated arrhythmia models, further expanding our ability to study and manipulate cardiac function.

## MATERIALS AND METHODS

### Mouse husbandry and breeding

All mouse work was conducted in accordance with Institutional Animal Care and Use Committee protocol 23-0020 of Washington University in St Louis. The Cre-Lox recombination system was used to generate transgenic mice with tissue-specific expression of the gene of interest. Specifically, LoxP-STOP-LoxP ReaChR reporter mice were crossed with a cardiac-specific Cre driver line to achieve heart-restricted expression. Strain #026294 B6.Cg-Gt(ROSA)26Sor^tm2.2Ksvo^/J (Rosa26 CAG-LSL-ReaChR-mCit), carrying the ReaChR and mCitrine transcription cassette, was obtained from the Jackson Laboratory. Strain #:024637 B6.129S1(SJL)-Nkx2-5tm2(cre)Rph/J (Nkx2.5-Cre), expressing an IRES-Cre fusion protein in cardiac crescent and heart tube progenitor cells, was also obtained from the Jackson Laboratory. All mice were housed at the Biomedical Engineering animal facility of Washington University in Saint Louis. They were maintained in a 12-hour light/12-hour dark cycle at 22°C, with freely available food and water.

Breeding colonies were set up with 4- to 12-week-old parents, and B6.Cg-Gt(ROSA)26Sor^tm2.2Ksvo^/J and B6.129S1(SJL)-Nkx2-5tm2(cre)Rph/J. F1 progeny (CAG-LSL-ReaChR-mCitrine/+; Nkx2.5-Cre/+) were genotyped by PCR. Animals with both transgenes present were subjected to pacing experiments. B6.Cg-Gt(ROSA)26Sor^tm2.2Ksvo^/+ siblings were used for control experiments.

### Polymerase chain reaction

Total genomic DNA was extracted from a mouse tail tissue fragment using a PureLink Genomic DNA Mini Kit (K182002, Thermo Fisher Scientific) according to the manufacturer’s instructions. PCR primers used to detect Rosa26 CAG-LSL-ReaChR-mCit were 5′-CTTCCCTCGTGATCTGCAAC-3′ and 5′-GTTATGTAACGCGGAACTCCA-3′. Primers for Nkx2.5-Cre detection were 5′-TTACGGCGCTAAGGATGACT-3′ and 5′-GTGTGGAATCCGTCGAAAGT-3′. DNA amplification was performed with GoTaq Green Master Mix (M7122, Promega), according to the manufacturer’s instructions.

### Heart slicing and staining

Dissected mouse hearts were washed in PBS and frozen through embedding in O.C.T. compound and placing over dry ice. Then, 10 μm thick sections were taken using a cryostat (CM1950, Leica) and stored at −80°C until staining. Slices were first defrosted, washed in PBS for 1 minute three times, fixed in 4% PFA for 12 min, washed in PBS for 1 min, three times; permeabilized with methanol for 6 min at −20°C; and washed again in PBS for 1 min, three times. The slices were mounted to slides using a mounting medium with DAPI (ProLongTM Diamond Antifade Mountant with DAPI, Invitrogen). Imaging was performed using a Zeiss Axioscan 7 slide scanner at ×20 magnification. DAPI was imaged with excitation centered at 385 nm, 5.2-ms exposure time, and a 430- to 470-nm emission filter. The endogenous mCitrine fluorophore was imaged with excitation centered at 475 nm, 60-ms exposure time, and 500- to 550-nm emission filter.

### Animal preparation

For all the noninvasive light stimulation experiments, the mice were anesthetized and maintained with isoflurane (1.0 to 2.0%) vaporized in medical air (1.75 liter/min) during all the procedures. The animals were positioned over a heating pad in a supine position. The hair on their chest was removed using a razor and Nair, exposing the chest skin and removing any effect of the hair’s melanin absorption on the recorded data.

### Area illumination for transthoracic light stimulation

Collimated light from a 590-nm or 617-nm LED (LCS-0590-03-11, LCS-0617-03-11, Mightex Inc.) illuminated the supine mouse chest perpendicularly. The LED optical power was delivered over an area of 5 or 10 mm in diameter on the chest surface. The LED output power and pulse width were controlled by an LED controller and software (Universal LED Controller, Mightex Inc.). The LED controller used an input signal from the ECG unit to trigger the light emission. For each experiment, the light stimulation period, frequency, pulse width, and power density were controlled to achieve the designed pacing protocol.

### ECG recording and analysis

Electrode needles were inserted subcutaneously into the animal limbs (Fig. 2B) and connected to the ECG unit (model ML865, PowerLab 4/25 T, AD Instruments Inc.). The ECG signal was recorded using LabChart 7.0 software (AD Instruments Inc.). The ECG unit provided an output signal to trigger light stimulation. The trigger signal was simultaneously recorded on ECG channel 1, allowing synchronization for data analysis.

To analyze the ECG data, we developed custom analysis code using Python and MATLAB. In Python, the ECG is subtracted from its baseline to avoid long-term drifting of the signal. The time position of the R peak of each heartbeat is identified, and the heart frequency is measured. The full width at half maximum (FWHM) of each heartbeat is measured before, during, and after stimulation. The capture array was built by setting a FWHM threshold at 5.5 ms to classify the heartbeat as captured or non-captured. The software also measures the time delay between the beginning of the light trigger signal and the R peak of the ECG. MATLAB software is used to measure the RR interval, which was used to calculate the HR over time. AI was defined for each interval as the standard deviation of the HR divided by the median HR.

### Cardiac optogenetic heart pacing

To demonstrate the feasibility of non-invasive mouse heart pacing at different frequencies, we used a pulsed light stimulation protocol and simultaneously recorded the ECG signal. The light illumination trigger was simultaneously recorded for further synchronization and analysis.

In tachypacing experiments shown in Figs. 2 and 3, we recorded the ECG trace before light stimulation for 15 s, followed by 10 s of light stimulation and 15 s after light stimulation to monitor the heart recovery, adding up to a total of 40 s. The irradiance and pulse width were determined by the strength-duration curve (rheobase and chronaxie). The stimulation frequency ranged from 6 to 14 Hz (360–840 bpm), with varying trials of 0.5 intervals in this range.

In Fig. 4, to evaluate the effect of longer pacing periods that competed with sinus rhythm, we recorded the ECG before light stimulation for 5 min, followed by 10 min of 617 nm light stimulation at 1.9 mW/mm^2^, and by 15 min after light stimulation to monitor the heart recovery, totaling 30 min. Light stimulation was performed with 6 Hz, 20-ms LPs; 0.1 Hz, 5-s LPs; and CW illumination, where the light was always on.

### Strength-duration curve

Cardiomyocyte action potential activation depends on the charge transferred across the cellular membrane, which in turn depends on the stimulus strength and duration. The strength-duration curve represents the threshold stimulus strength (power density) necessary to pace the heart at a given stimulus duration.

After animal preparation, we measured the strength-duration curve of 6 mice, with stimulation at 590 and 617 nm. The stimulus pulse widths ranged from 2.5 to 100 ms, and the threshold strength needed for excitation was recorded. The strength-duration curve asymptotically converged to a plateau value, known as the rheobase. After that, the chronaxie, the stimulus duration where the stimulus strength is twice the rheobase, was determined.

### Capture rate analysis

The capture rate is the percentage of the heart contraction initiated by a light pulse over all heartbeats. A light-induced contraction is defined here by the elongated QRS complex duration, indicating that the heartbeat initiation is not from the SN. We used the FWHM of the QRS complex to classify the heartbeats and quantify the wider QRS complex (fig. S3). Based on experimental observation, a heartbeat was categorized as light-induced if the length of the QRS complex was above a threshold value of 5.5 ms.

### MC simulation

MC light propagation simulations were performed using the MCmatlab program developed by Marti *et al.* (*52*) The model geometry was defined as a 30 mm–by–30 mm–by–30 mm cube of homogenous media of mouse skin at 617 nm (μ’_s_ of 90 cm^−1^, μ_a_ of 0.9 cm^−1^, *g* = 0.9, and *n* = 1.37) or mouse skin at 590 nm (μ’_s_ of 90 cm^−1^, μ_a_ of 1.1 cm^−1^, *g* = 0.9, and *n* = 1.37) (*44*, *52*). The simulation was run using 1 × 10^9^ photons. The single fiber/LED illumination was represented using an isotropic point source, with area of 0.01 mm^2^. The area illumination was represented by a Lambertian distribution with radius of 5 or 10 mm.

### Echocardiogram

To directly visualize and measure the heart function and hemodynamic changes induced by the light pacing, we imaged the heart using an Vevo 3100 ultrasound (US) system (FUJIFILM VisualSonics Inc.). We placed the mouse (*n* = 4) supine on a heating pad, with its arms and legs fixed to the ECG electrodes. We placed the US probe over the mouse’s chest using acoustic gel and positioned it to acquire B-mode cine record from the long axis LV and pulsed-wave Doppler mode records from the AA. Simultaneously, we irradiated the mouse’s chest over the heart location with a collimated LED light. We obtained video records for ~25 s at 259 frames per second. For both the LV and AA, we divided the recording time into three sections: 5 s of resting, 10 s of light stimulation, and 10 s of recovery. We stimulated the heart using continuous illumination/wave (CW), LP (0.1 Hz), and 6-, 8-, 10-, and 14-Hz frequencies.

The FS was calculated by

$$\text{FS}\left(\%\right)=\frac{\text{LVIDd}-\text{LVIDs}}{\text{LVIDd}}\times 100\%$$

where LVIDd and LVIDs represent the LV internal dimensions (LVIDs) at the diastole and systole stages, representatively. They were measured from the M-mode images.

The CO is calculated by

CO=VTI×Area×HR

where VTI denotes the integration of the blood flow velocity over time, Area represents the aorta cross-sectional area, and HR stands for HR. From PW Doppler images of the AA, we obtained the blood flow velocity during the cardiac cycle and integrated the VTI over one second, which is the equivalent of the multiplying the VTI and HR. The aorta’s cross-sectional area was recorded in a snapshot visualizing the LV aorta separate from the video recording. Using Vevo Lab software (FUJIFILM VisualSonics Inc.), we performed all measurements at 1 s intervals at six different time intervals: 1.0, 2.5, and 4.0 s during resting, 5.5, 9.0, and 14.5 s during stimulation, and 16.0, 17.5, and 24.5 s during recovery intervals.

### Optical intrinsic signal imaging

A custom light engine consisting of 530-nm (measured peak λ = 525 nm, LCS-0525-60-22, Mightex Systems) and 625-nm (measured peak λ = 637 nm, M625L3, Thorlabs) LEDs illuminated the skull (Fig. 5A). Diffuse-reflected light for optical intrinsic signal was collected by a lens (focal length = 75 mm, NMV-75 M1, Navitar), split by a 580-nm dichroic (FF580-FDi02-t3-25x36, Semrock) and sampled by two scientific complementary metal-oxide semiconductor (CMOS) cameras with USB3 connectivity (Zyla 5.5, Andor). A 500-nm long-pass filter (FF01-500/LP-25, Semrock) in front of camera 1 (CMOS1) passed 530-nm reflectance. The 580-nm dichroic and a 593-nm long-pass filter (FF01-593/LP-25, Semrock) in front of camera 2 (CMOS2) passed 625-nm reflectance. Each camera sensor was cropped to 1024 × 1024 pixels, and 2 × 2 binning was performed on camera to increase the acquisition rate. The frame rate of each camera was 100 Hz, and all contrasts were imaged at 20 Hz. The LEDs and camera exposures were synchronized and triggered via a data acquisition card (PCI-6733, National Instruments) using MATLAB R2019a (MathWorks). The pulse durations for the LEDs were 0.2 ms (530-nm reflectance) and 0.3 ms (625-nm reflectance). LED spectra were measured using an Ocean Optics USB 200+ spectrometer.

### Imaging acquisition

Mice were mounted to the imaging system as per previous reports (*53*) using an aluminum bracket attached to the skull-mounted Plexiglas window. Mice were anesthetized and maintained with isoflurane (<1.0%) vaporized in medical air (1.5 liter/min) during all imaging. The animals were positioned under a heating pad, and the body was supported by a felt pouch suspended by steel posts with an opening for heart pacing. Resting state imaging was performed for 10 min in each mouse before heart pacing. Heart pacing was performed as previously described in 30-s blocks, 5 s on followed by 25-s rest, for a total of 5 min followed by a 5-min rest from heart pacing. This was repeated for each pacing frequency (6, 8, 10, and 14 Hz). Before each imaging run, dark counts due to background sensor noise were imaged for 1 s with both LEDs off.

### OISI data analysis

#### Spatial normalization

Images (512 × 512 pixels after on-camera binning) were binned 4 × 4 off camera (to a resolution of 128 × 128) to increase the signal-to-noise ratio of each image and to reduce data size. To coregister image sequences from each camera, four corresponding markers were chosen from images of 530 nm reflectance (CMOS1) and 625 nm reflectance (CMOS2) and spatially normalized using a projective transformation to account for slight differences in camera alignment and fields-of-view. Dark frames from each camera were averaged and subtracted from that camera’s image sequence. All image sequences were then affine transformed and aligned to the Allen mouse brain atlas as per previous studies (*53*).

#### Image processing

Slow trends in light level (e.g., due to fluctuations in LED illumination) were temporally detrended using a fifth-order polynomial fit. The images were spatially smoothed with a Gaussian filter (5 × 5 pixel kernel with an SD of 1.3 pixels). Changes in 530-nm and 625-nm reflectance were interpreted using the modified Beer-Lambert law to calculate changes in hemoglobin concentration as per previous studies (*54*, *55*)

$$\Phi \left(r,t\right)=\Phi_{0}e^{-\Delta \mu_{\alpha }\left(r,t\right)L}$$

where Φ(r,t) is the measured light intensity, $\Phi_{0}$ is the baseline light intensit*y*, $-\Delta \mu_{\alpha }\left(r,t\right)$ is the change in absorption coefficient due to changes in hemoglobin concentration, and L is the optical path length factor for photons traveling in tissue determined analytically assuming a semi-infinite geometry (*56*). Assumed optical properties are the same as per previous reports (*53*). Changes in hemoglobin concentration were determined by inverting the system of equations

$$\Delta \mu_{\alpha ,\lambda }\left(r,t\right)=E_{\lambda ,i}\Delta \left[Hb_{i}\left(r,t\right)\right]$$

where E is the extinction coefficient for hemoglobin and i is the index for different hemoglobin species.

### Heart pacing evoked activity

Image sequences of heart pacing evoked activity were averaged across pacing presentations and then across mice. Images during the pacing period were averaged to generate group-level maps of peak response for each contrast. Time courses were calculated by averaging all time courses in all pixels.

### Statistical analysis

For comparisons on echocardiographic measurements, a repeated-measures ANOVA was first applied to check for differences between resting and pacing phases, pacing conditions, and the interaction between pacing phase and condition. If the ANOVA comparing resting and pacing was significant, then a Wald-type *t* test was performed for post hoc pairwise comparisons between groups. For all other comparisons, a one-way ANOVA was first applied to determine whether differences existed between groups. The independent variable was the pacing stimulus applied, and the dependent variable was the measured response. If the ANOVA was significant, then a Tukey’s post hoc test was applied for post hoc pairwise comparisons between groups. Results were deemed significant when *P* < 0.05.
