# Supplementary material for: Noninvasive optogenetic induction of cardiac arrhythmias alters systemic hemodynamics in mice
Source: Sci Adv. 2026 Jun 3;12(23):eaeb1092. doi: 10.1126/sciadv.aeb1092 (PMC13232562; doi:10.1126/sciadv.aeb1092)
Supplement: Supplementary file 1 — Figs. S1 to S11 Legends for movies S1 to S12 Legend for supplementary data [file sciadv.aeb1092_sm.pdf]

Science Advances
AAAS

Supplementary Materials for

Noninvasive optogenetic induction of cardiac arrhythmias alters systemic hemodynamics in mice

Marcello Magri Amaral *et al.*

Corresponding author: Chao Zhou, chaozhou@wustl.edu

Sci. Adv. **12**, eaeb1092 (2026)
DOI: 10.1126/sciadv.aeb1092

The PDF file includes:

Figs. S1 to S11
Legends for movies S1 to S12
Legend for supplementary data

Other Supplementary Material for this manuscript includes the following:

Movies S1 to S12
Supplementary Data

Supplementary Figures

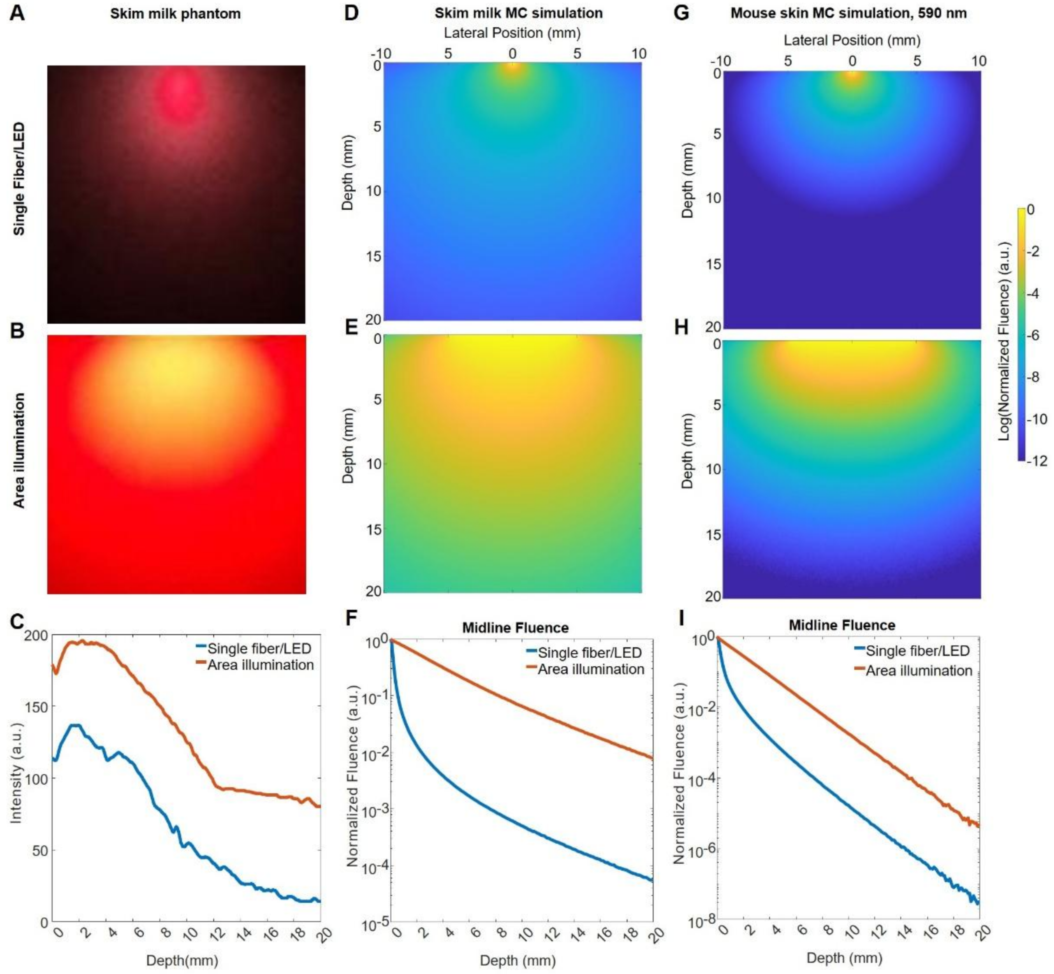

Fig. S1: Spot size affects pacing efficiency and wavelength-dependent light penetration. (A-B) Scattering experimental results, using skim milk as a scattering medium, for optical fiber (A) and area illumination (B; 10 mm spot diameter) light delivery comparison at 617 nm. (**C**) Intensity profile at the midline from the optical fiber (A) and area illumination (B) scattering experiment. (**C-D**) Monte-Carlo (MC) simulation results for 617 nm light delivered by a single fiber/LED (D) and a large area illumination (E; 10 mm spot size) propagating through a skim milk scattering medium. (**F**) Normalized fluence at the middle of the single fiber/LED (D) and area illumination (E) from the MC simulation for the skim milk scattering medium. **(G-H)** Monte-Carlo (MC) simulation results for 590 nm light delivered by a single fiber/LED (G) and area illumination (H; 10 mm spot size) propagating through the skin scattering medium. (**I**) Normalized fluence at the midline from the single fiber/LED (G) and area illumination (H) MC simulation for the skin scattering medium. The MC simulation and the scattering experimental results show that by using the same power density at the medium surface, the total power delivered at each depth position is higher when using the area illumination approach compared to the single fiber delivery. Moreover, the power distribution in the lateral direction is wider using area illumination which increases the pacing likelihood and reduces mispositioning of the light stimulation. This large illumination approach is especially important to pace larger animals and maintain the power density below the minimum permissible exposure for the skin.

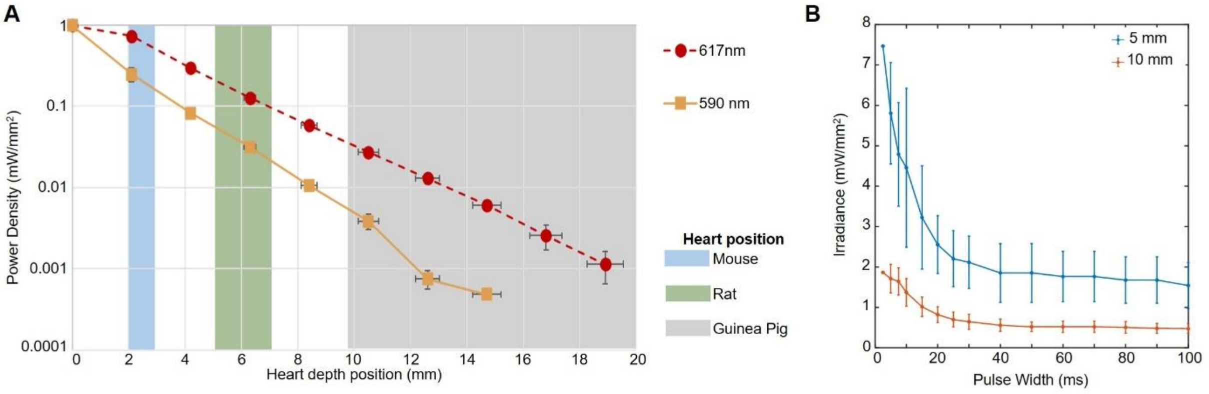

Fig. S2: Light penetration and strength-duration curves for different spot diameters. (**A**) Normalized power density attenuation on a chicken breast tissue phantom for 590 and 617 nm comparisons. The power density attenuation in the tissue phantom shows that 617 nm penetrates deeper into the tissue than 590 nm. The shadowed colors represent the heart depth of different animals (blue, mouse; green, rat; gray; guinea pig). Using longer wavelengths can represent about one order of magnitude smaller attenuation for larger animal studies. (**B**) Strength-duration curves using 617 nm light for 5- and 10-mm spot diameters (n = 14 (n=3 female and n=11 male) and n = 5 (n=3 female and n=2 male), respectively). A larger spot size requires less power density to pace the heart.

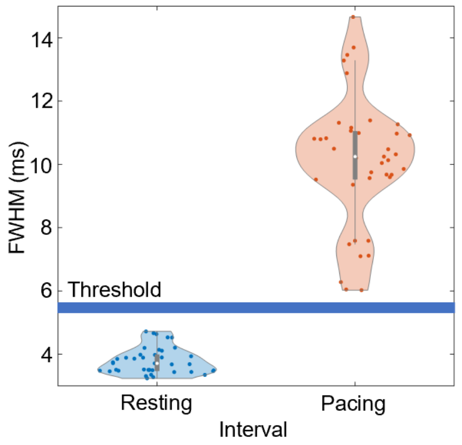

Fig. S3: Light-induced heartbeat classification. Full width at half maximum (FWHM) of the QRS complex for resting and pacing intervals in one recording. The FWHM values of the QRS complex due to the SN rhythm (resting) are smaller than those during light pacing. The broadening in the QRS complex observed during pacing was used to automatically classify each heartbeat as captured (light pacing) or non-captured (SN rhythm). The threshold for determining light pacing is a 5.5 ms QRS complex FWHM. Measuring the FWHM is advantageous over direct measurement of the QRS interval for automated classification because it is more precise and less susceptible to the influence of noise.

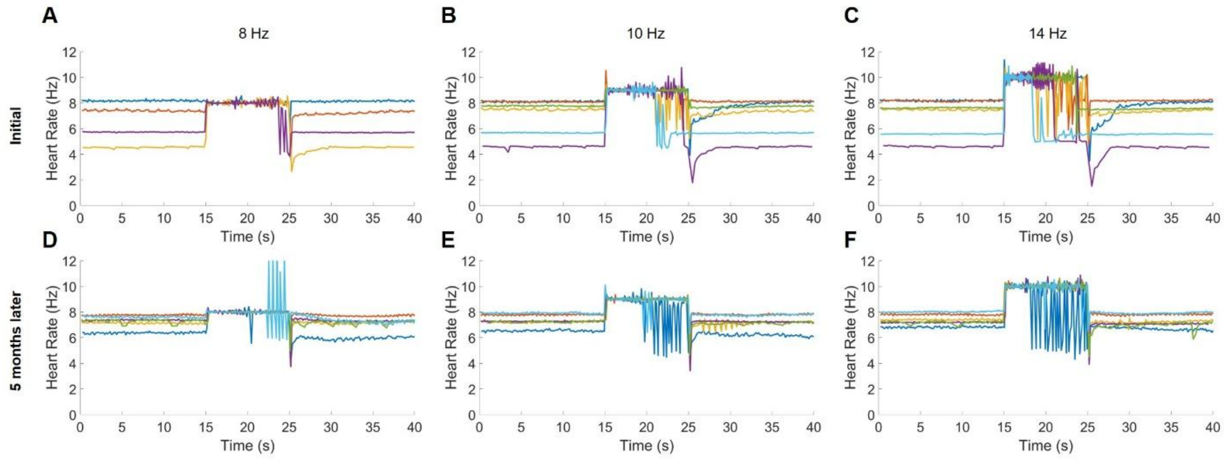

Fig. S4: Stable opsin expression allows for pacing throughout the life of the mouse. (**A-C**) initial HR and (**D-F**) HR of transgenic mice from 5 months later (n = 6, n=1 female and n=5 male) for 8, 9 and 10 Hz. The opsin expression in transgenic mice is stable over time, and the same mice can be paced at different time points with the same pacing efficiency.

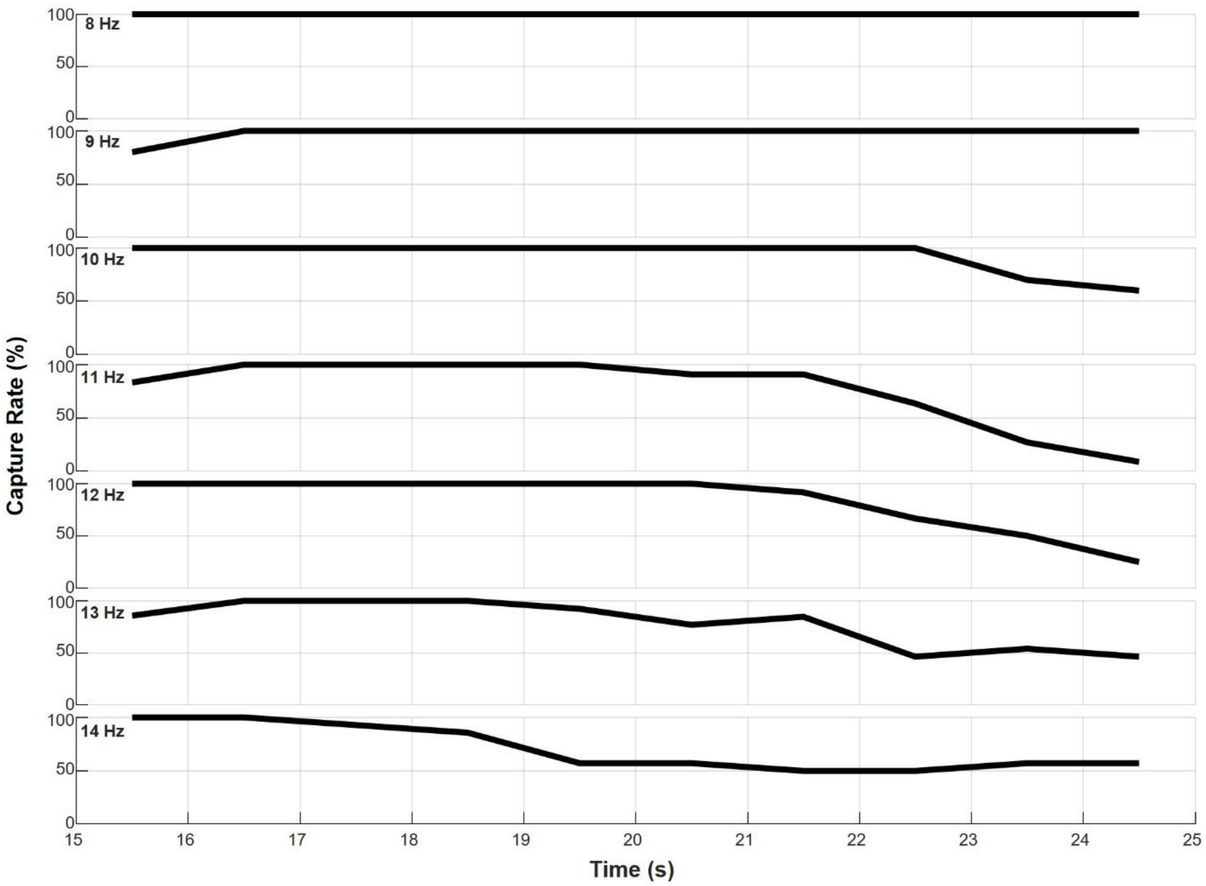

Fig. S5: Representative capture rate results for light-induced tachycardia from 8 to 14 Hz pacing. Increasing the pacing frequency reduces the capture rate over time and decreases the time during which the capture rate can be sustained at a 100 % level. For this particular case, the capture rate for 14 Hz pace decreased to 50 %, which corresponds to a 7 Hz heartbeat.

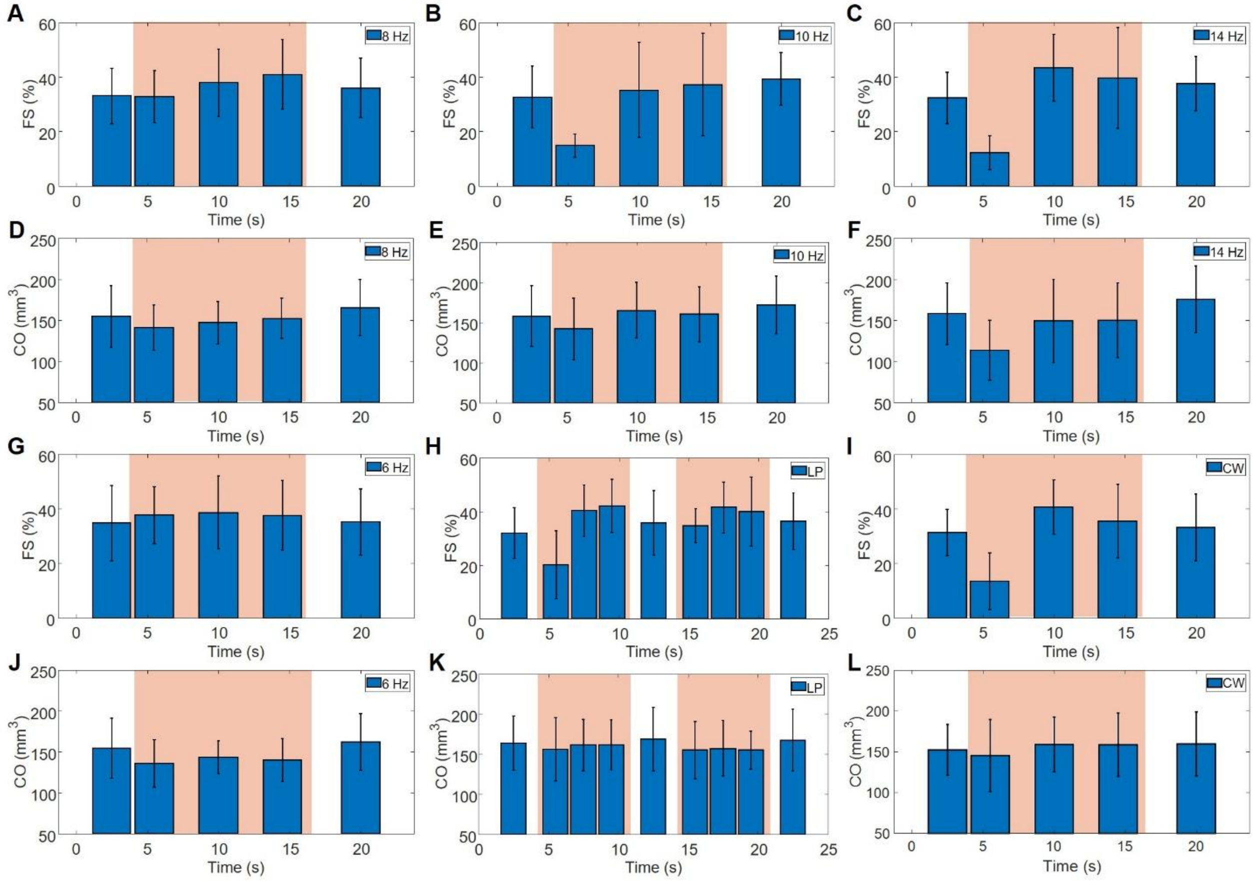

Fig. S6: Changes in fractional shortening (FS) and cardiac output (CO) changing during pacing. (**A-C**) FS for 8, 10, 14 Hz pacing. (**D-F**) CO for 8, 10, and 14 Hz pacing. (**G-I**) FS for the 6 Hz, long pulse (LP), and CW light arrhythmia-inducing protocols. (**J-L**) CO for the 6 Hz, long pulse (LP), and CW light arrhythmia-inducing protocols. The shaded area represents the light stimulation interval. Plots reflects n=4 total (n=2 female and n=2 male) mice.

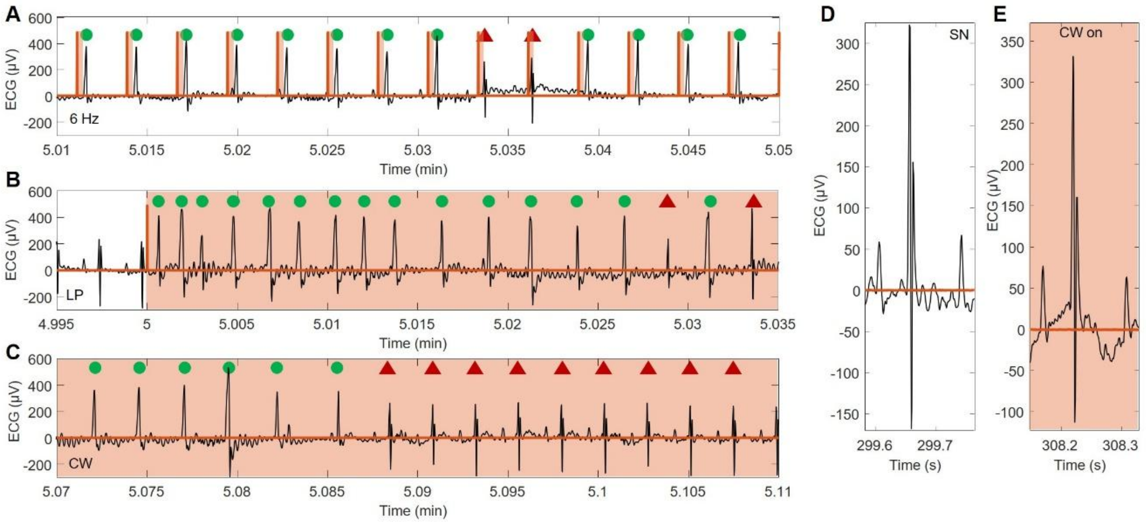

Fig. S7: Electrocardiograms (ECG) of light-induced arrhythmia. ECG for the (**A**) 6 Hz, (**B**) LP, and (**C**) CW stimulation protocols. The shaded area represents the period of light stimulation. For the 6 Hz stimulation protocol, the pacing frequency is slower than the RHR, which creates competition between the SN and light pacing rhythm, increasing the arrhythmia during pacing. For the LP, the increased arrhythmic heart function is due to the creation of an irregular HR during the 1 s of light illumination. For the CW protocol, a change in HR is observed at the beginning of the light illumination, returning to its SN rhythm after a few heart cycles. Despite returning to the SN rhythm, compared to the SN rhythm during resting period (**D**), a change in the ECG morphology is observed during light illumination (**E**). Typical ECG of a single heartbeat due to (D) the SN rhythm and (E) CW stimulation. Green dots denote light captured beats, while red triangles represent beats due to sinus rhythm.

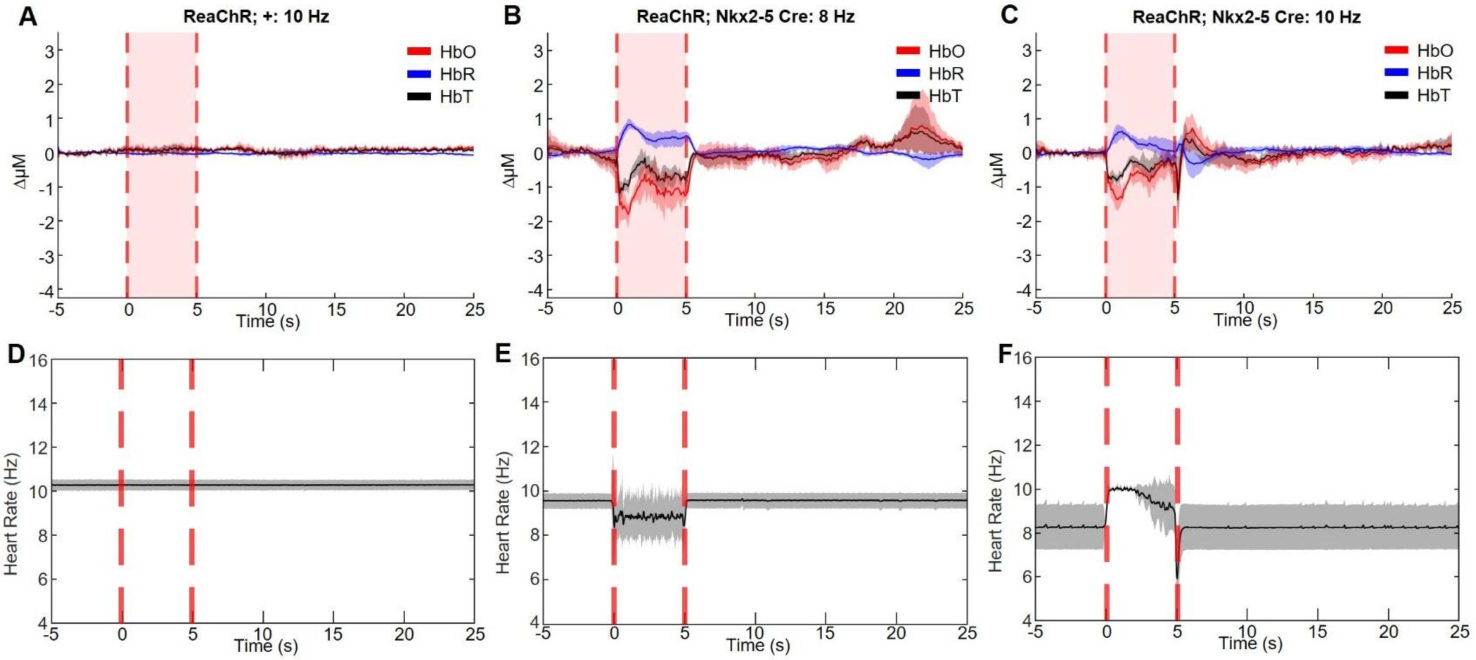

Fig. S8: Timewise plots of changes in hemoglobin contrasts and heart rate. (A) Changes in oxygenated (red), deoxygenated (blue), and total hemoglobin (black) concentration in the control group with 10 Hz light stimulation, **(B)** experimental group with 8 Hz light stimulation, and **(C)** experimental group with 10 Hz light stimulation. **(D)** Changes in heart rate in the control group with 10 Hz light stimulation, **(E)** experimental group with 8 Hz light stimulation, and **(F)** experimental group with 10 Hz light stimulation. Shading around each contrast is plotted as +/- standard deviation. The experimental group reflects n=5 total (n=3 female and n=2 male) mice. The control group reflects n=5 total (n=1 female and n=4 male) mice.

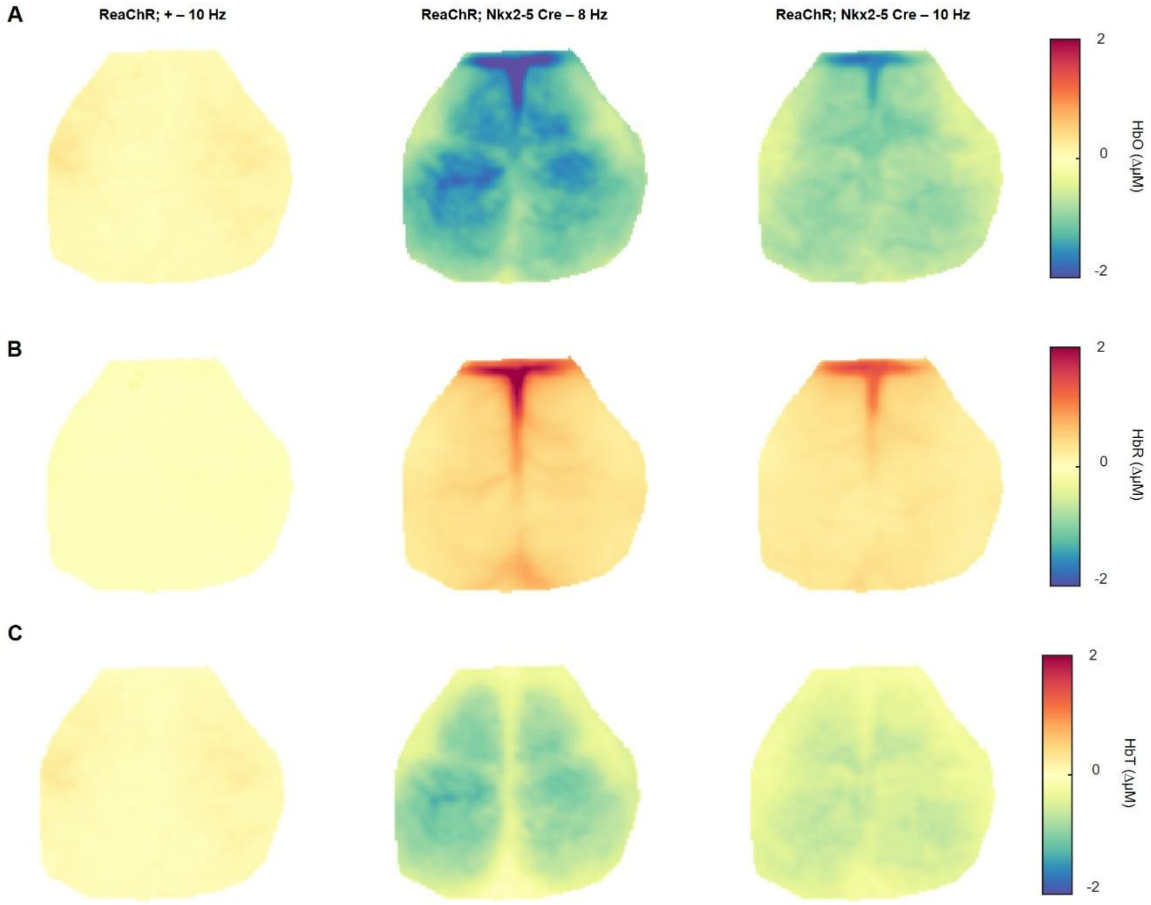

Fig. S9: Experimental and control group peak maps during light stimulation. (**A)** Oxygenated hemoglobin peak maps, **(B)** deoxygenated hemoglobin peak maps, and **(C)** total hemoglobin peak maps for control group (light stimulation at 10 Hz), and experimental group (light stimulation at 8 Hz and 10 Hz). The experimental group reflects n=5 total (n=3 female and n=2 male) mice. The control group reflects n=5 total (n=1 female and n=4 male) mice.

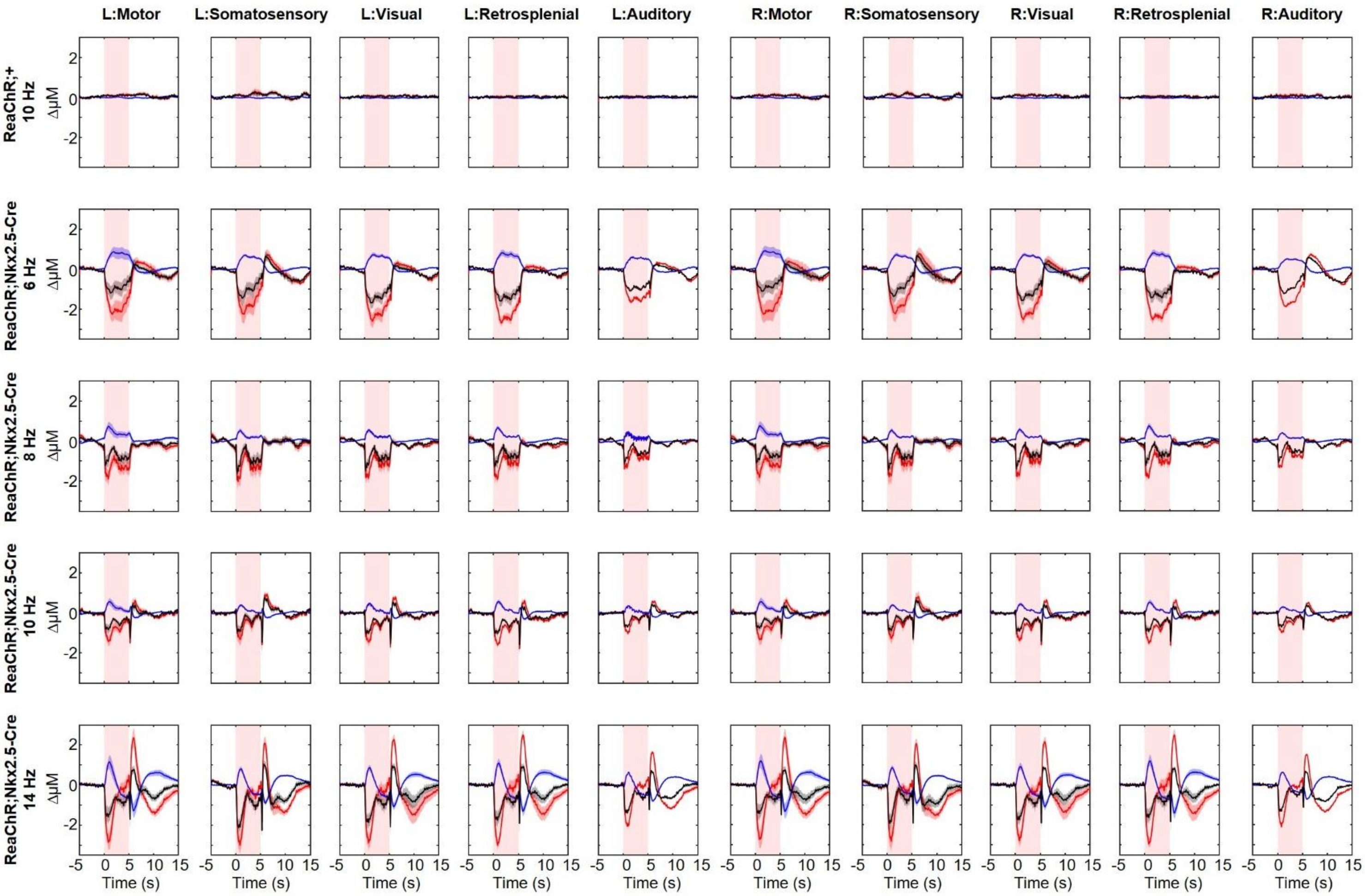

Fig. S10: Hemodynamic changes across different cortexes. The cortex is parceled according to the Allen atlas into left (L) and right (R) motor, somatosensory, visual, Retrosplenial, and auditory cortexes. Changes in oxygenated (red), deoxygenated (blue), and total (black) hemoglobin are plotted for each pacing frequency with shading representing +/- standard deviation. The experimental group reflects n=5 total (n=3 female and n=2 male) mice. The control group reflects n=5 total (n=1 female and n=4 male) mice.

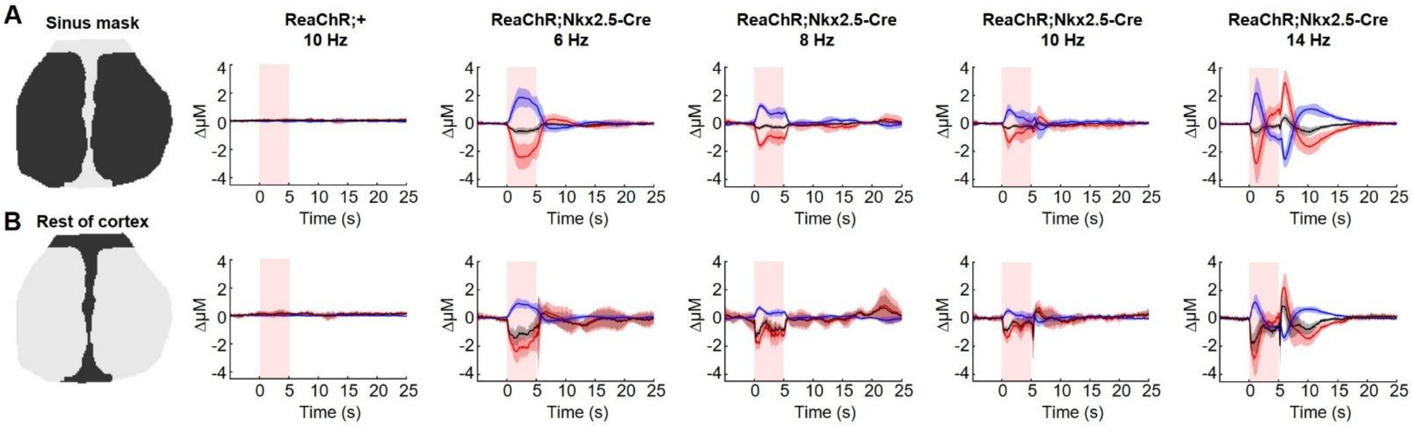

Fig. S11: Sinus distinct dynamics. A) Mask of superior sagittal sinus with average changes in oxygenated (red), deoxygenated (blue), and total hemoglobin (black) concentrations during pacing in the superior sagittal sinus masked in (A). **(B)** Mask of rest of the cortex, excluding superior sagittal sinus with average changes in oxygenated, deoxygenated, and total hemoglobin concentrations during pacing in the superior sagittal sinus masked in (B). Each contrast is plotted as the mean with shaded regions indicating +/- standard deviation. All group analysis in this figure was performed with n=5 samples for experimental and control groups. The experimental group reflects n=5 total (n=3 female and n=2 male) mice. The control group reflects n=5 total (n=1 female and n=4 male) mice.

Supplementary Movie 1: Supplementary Movie 1 contains a demonstration of tunable tachycardia. The top panel displays the ECG signal, the middle panel plots the light pulse signal, and the bottom panel shows the heart rate. 15 seconds of rest are observed before pacing at 10 Hz, 8 Hz, 11 Hz, 9 Hz, and 9.5 Hz in sequence.

Supplementary Movie 2: Supplementary Movie 2 contains a demonstration of ECG and heart rate response to 8 Hz light stimulation. The top panel displays the ECG signal, the middle panel plots the light pulse signal, and the bottom panel shows the heart rate.

Supplementary Movie 3: Supplementary Movie 3 contains a demonstration of ECG and heart rate response to 10 Hz light stimulation. The top panel displays the ECG signal, the middle panel plots the light pulse signal, and the bottom panel shows the heart rate.

Supplementary Movie 4: Supplementary Movie 4 contains a demonstration of ECG and heart rate response to 14 Hz light stimulation. The top panel displays the ECG signal, the middle panel plots the light pulse signal, and the bottom panel shows the heart rate.

Supplementary Movie 5: Supplementary Movie 5 shows an echocardiogram recording of the left ventricle during 8 Hz light stimulation, slowed down to 0.1x speed. The bottom portion of the screen plots the ECG signal in green, respiration in yellow, and light pulses in red.

Supplementary Movie 6: Supplementary Movie 6 shows an echocardiogram recording of the left ventricle during 10 Hz light stimulation, slowed down to 0.1x speed. The bottom portion of the screen plots the ECG signal in green, respiration in yellow, and light pulses in red.

Supplementary Movie 7: Supplementary Movie 7 shows an echocardiogram recording of the left ventricle during 14 Hz light stimulation, slowed down to 0.1x speed. The bottom portion of the screen plots the ECG signal in green, respiration in yellow, and light pulses in red.

Supplementary Movie 8: Supplementary Movie 8 displays changes in cerebral hemodynamics during 6 Hz light stimulation. The top row shows masks of the cortex which show the spatial distribution of changes in oxygenated hemoglobin (left), deoxygenated hemoglobin (middle), and total hemoglobin (bottom). The bottom panel plots the heart rate.

Supplementary Movie 9: Supplementary Movie 9 displays changes in cerebral hemodynamics during 8 Hz light stimulation. The top row shows masks of the cortex which show the spatial distribution of changes in oxygenated hemoglobin (left), deoxygenated hemoglobin (middle), and total hemoglobin (bottom). The bottom panel plots the heart rate.

Supplementary Movie 10: Supplementary Movie 10 displays changes in cerebral hemodynamics during 10 Hz light stimulation. The top row shows masks of the cortex which show the spatial distribution of changes in oxygenated hemoglobin (left), deoxygenated hemoglobin (middle), and total hemoglobin (bottom). The bottom panel plots the heart rate.

Supplementary Movie 11: Supplementary Movie 11 displays changes in cerebral hemodynamics during 14 Hz light stimulation. The top row shows masks of the cortex which show the spatial distribution of changes in oxygenated hemoglobin (left), deoxygenated hemoglobin (middle), and total hemoglobin (bottom). The bottom panel plots the heart rate.

Supplementary Movie 12: Supplementary Movie 12 displays changes in cerebral hemodynamics during 10 Hz light stimulation in a control mouse (ReaChR; +). The top row shows masks of the cortex which show the spatial distribution of changes in oxygenated hemoglobin (left), deoxygenated hemoglobin (middle), and total hemoglobin (bottom). The bottom panel plots the heart rate.

Supplementary Data: The supplementary data file contains all data underlying the bar plots and statistical analysis in the results, labeled by figure subpanel.
